# MEK inhibition drives anti-viral defence in RV but not RSV challenged human airway epithelial cells through AKT/p70S6K/4E-BP1 signalling

**DOI:** 10.1186/s12964-019-0378-7

**Published:** 2019-07-18

**Authors:** Engin Baturcam, Stefan Vollmer, Holger Schlüter, Rose A. Maciewicz, Nisha Kurian, Outi Vaarala, Stephan Ludwig, Danen Mootoosamy Cunoosamy

**Affiliations:** 1 Early Respiratory, Inflammation & Autoimmunity, R&D BioPharmaceuticals, AstraZeneca, Gothenburg, Sweden; 2 Precision Medicine, R&D Oncology, AstraZeneca, Gothenburg, Sweden; 3 Early Respiratory, Inflammation & Autoimmunity, R&D BioPharmaceuticals, Gaithersburg, USA; 4 Institute of Virology Muenster, Westfaelische Wilhelms-University Muenster, Muenster, Germany

**Keywords:** Human rhinovirus, Respiratory syncytial virus, Innate immune response, Interferon-β, Interferon stimulated genes, Dual specificity mitogen-activated protein kinase kinase (MEK) pathway, Phosphoinositide-3-kinase (PI3K) pathway, Primary airway epithelial cells

## Abstract

**Background:**

The airway epithelium is a major target tissue in respiratory infections, and its antiviral response is mainly orchestrated by the interferon regulatory factor-3 (IRF3), which subsequently induces type I (β) and III (λ) interferon (IFN) signalling. Dual specificity mitogen-activated protein kinase kinase (MEK) pathway contributes to epithelial defence, but its role in the regulation of IFN response in human primary airway epithelial cells (AECs) is not fully understood. Here, we studied the impact of a small-molecule inhibitor (MEKi) on the IFN response following challenge with two major respiratory viruses rhinovirus (RV2) and respiratory syncytial virus (RSVA2) and a TLR3 agonist, poly(I:C).

**Methods:**

The impact of MEKi on viral load and IFN response was evaluated in primary AECs with or without a neutralising antibody against IFN-β. Quantification of viral load was determined by live virus assay and absolute quantification using qRT-PCR. Secretion of cytokines was determined by AlphaLISA/ELISA and expression of interferon-stimulated genes (ISGs) was examined by qRT-PCR and immunoblotting. A poly(I:C) model was also used to further understand the molecular mechanism by which MEK controls IFN response. AlphaLISA, siRNA-interference, immunoblotting, and confocal microscopy was used to investigate the effect of MEKi on IRF3 activation and signalling. The impact of MEKi on ERK and AKT signalling was evaluated by immunoblotting and AlphaLISA.

**Results:**

Here, we report that pharmacological inhibition of MEK pathway augments IRF3-driven type I and III IFN response in primary human AECs. MEKi induced activation of PI3K-AKT pathway, which was associated with phosphorylation/inactivation of the translational repressor 4E-BP1 and activation of the protein synthesis regulator p70 S6 kinase, two critical translational effectors. Elevated IFN-β response due to MEKi was also attributed to decreased STAT3 activation, which consequently dampened expression of the transcriptional repressor of *IFNB1* gene, PRDI-BF1. Augmented IFN response translated into inhibition of rhinovirus 2 replication in primary AECs but not respiratory syncytial virus A2.

**Conclusions:**

Our findings unveil MEK as a key molecular mechanism by which rhinovirus dampens the epithelial cell’s antiviral response. Our study provides a better understanding of the role of signalling pathways in shaping the antiviral response and suggests the use of MEK inhibitors in anti-viral therapy against RV.

**Electronic supplementary material:**

The online version of this article (10.1186/s12964-019-0378-7) contains supplementary material, which is available to authorized users.

## Background

The airway epithelium is directly exposed to respiratory viruses and provides a first-line of defence as a major component of the innate immune response system. This early immune response, which consists of inflammatory cytokines and interferons (IFN), is triggered upon infection by respiratory viruses [1], including rhinovirus (RV) or respiratory syncytial virus (RSV) both known to cause exacerbations in chronic lung diseases such as asthma and chronic obstructive pulmonary disease (COPD) [2]. Several pattern recognition receptors (PRRs) in the airway epithelium are engaged during respiratory viral infection including endosomal toll-like receptor 3 (TLR3), cytosolic retinoic acid inducible gene I (RIG-I) and melanoma differentiation-associated gene 5 (MDA5) [1]. PRR activation in airway epithelial cells (AECs) results in the induction of type I (primarily IFN-β) and type III (IFN-λ1, 2, and 3) IFNs as well as an inflammatory cytokine response. Interferon regulatory factor 3 (IRF3) is a transcription factor that is constitutively expressed in AECs and plays an important role in mounting a rapid IFN response following viral infection [3]. IRF3 is activated following phosphorylation by tank binding kinase 1 (TBK1) and inhibitor-κB kinase ε (IKKε) [4, 5], which leads to homo or hetero-dimerization of IRF3 and its translocation to the nucleus where it activates transcription of *IFNB1* and *IFNLs* genes. The phosphoinositide-3-kinase/protein kinase B (PI3K/AKT) pathway is a critical determinant of type I IFN response and the recruitment of PI3K following TLR3 and RIG-I activation significantly contributes to IRF3 activation and subsequent transcriptional activation of IFNs [6–9]. In addition, it has been proposed that an interaction between AKT and TBK1 is essential for optimal activation of IRF3 [10].

Secreted IFN-β and IFN-λ engage IFN-α receptor (IFNAR) and IFN-λ receptor (IFNλR), respectively, leading to activation of the receptor-associated protein tyrosine kinases janus kinase 1 (JAK1) and tyrosine kinase 2 (TYK2). These kinases phosphorylate cytoplasmic transcription factors signal transducer and activator of transcription 1 (STAT1) and STAT2, enabling their dimerization. Upon translocation to the nucleus, activated STAT1-STAT2 dimers form a complex with IFN-regulatory factor 9 (IRF9) called IFN-stimulated gene factor 3 (ISGF3) and activate transcription of interferon stimulated genes (ISGs).

A rapid IFN response is critical to containing viral infections and an augmented innate immunity, without the deleterious effects of an excessive response, and may represent a pertinent strategy for fighting viral infections. In addition to viruses, several host factors have been identified as key regulators of the IFN signaling, demonstrable at both transcriptional level and translational levels [11].

Both RV and RSV promote activation of the epidermal growth factor receptor (EGFR) and its subsequent signaling involving the rapidly accelerated fibrosarcoma / dual specificity mitogen-activated protein kinase kinase / extracellular signal-regulated kinases (RAF/MEK/ERK) axis. Previous work suggested a possible involvement of the MEK pathway in the regulation of the IFN response. A crosstalk between ERK and IFN pathways has been identified in macrophages from mice, although the molecular mechanism leading to an increased IFN response following lipopolysaccharide-induced TLR4 stimulation is yet to be described [12]. However, the interaction between MEK and IFN pathways in the context of TLR3 and RIG-I signaling in the airway epithelium has not been studied. Here, we investigated the role of MEK pathway in regulating IFN response in human airway epithelial cells and we show that pharmacological inhibition of MEK enhances type I IFN response through improved translation control of IRF3-driven innate immunity and reduction of the *IFNB1* gene repressor, positive regulatory domain I-binding factor1 (PRDI-BF1) but only with RSVA2 infection and not with RV2. As a result, this translates into reduction of RV2 load in AECs but not that of RSVA2, suggesting that RSVA2-specific factors antagonize the beneficial effects of MEK inhibition.

## Methods

### Subjects

NHBE cells from 6 human donors were commercially available from Lonza AG. The median age was 33 years, range (2–65) and the population was equally split between males and females. All donors had no history of smoking (Additional file 1: Table S1).

### Primary culture and cell line

AECs were initially seeded into 25 cm^2^ cell culture flasks in steroid-supplemented bronchial epithelial growth medium (BEGM; Lonza AG). Following the second passage, cells were seeded into 25 cm^2^ cell culture flasks (60,000 cells / flask), 12-well (30,000 cells / well) or 96-well (10,000 cells / well) plates and cultured as submerged monolayers until 75% confluent [13]. The culture medium was then replaced with steroid-free medium 24h prior to treatment with MEKi (10 nM for 1h) (Additional file 1: Table S2) or DMSO control. Cells were then stimulated with poly(I:C) or infected with RV2 or RSVA2.

H1-HeLa cells (ATCC, CRL-1958™) were grown in minimum essential media supplemented with glutamine, non-essential amino acids, and 10% foetal bovine serum (complete MEM). Cells were treated with MEKi (10 nM for 1h) and infected with RV2.

BEAS-2B cells (ATCC, CRL-9609™) were grown in Dulbecco’s modified Eagle’s medium nutrient mixture F-12 (DMEM F12), supplemented with 10% foetal bovine serum and antibiotics (100 Units/ml penicillin and streptomycin). Cells were treated with MEK inhibitors (parental small molecule inhibitor or the MEK PROTAC (10 μM for 16h) and stimulated with poly(I:C) for 4h.

### Virus strains

High titer stocks of RV2 (lot K1227B) and RSVA2 (Lot I1629A) were commercially available from Virapur. All the viruses were stored at − 80 °C.

### Infection with RV2 or RSVA2

AECs cultured in 12-well plates were infected with either RV2 or RSVA2 at a multiplicity of infection (MOI) of 0.1 plaque-forming units (pfu)/cell. Viruses were inactivated by UV light irradiation (1200 mJ/cm2 for 30 min). Uninfected control cells were exposed to virus-free media. Cells were incubated with virus for 2h at 37 °C, and then washed three times with 1X PBS after which steroid-free culture medium (BEGM; Lonza) was added in presence or absence of a neutralizing anti-IFN-β antibody (0,2 μg/ml, R&D AF814). After 24h post-infection (p.i.), cells were lysed either for total RNA or protein extraction. Supernatants were stored for cytokine quantification, LDH assay and live virus assay.

H1-HeLa cells cultured in 12-well plates were infected with RV2 at a MOI of 0.1. Cells were incubated with virus for 2h at 37 °C, and then washed three times with 1X PBS after which complete MEM was added. Samples were collected 24h p.i.

### Stimulation with poly(I:C)

AECs cultured in 96-well or 12-well plates were stimulated with poly(I:C) (10 μg/ml, Sigma P1530). Cells were sampled at indicated time points within 24h post-treatment (p.t.) for RNA extraction, SureFire® assay or immunofluorescence. Supernatant was collected for cytokine measurements.

### Preparation of whole cell extracts and subcellular fractionation

Whole cell extracts for SDS-PAGE based runs were obtained using RIPA buffer supplemented with phosphatase inhibitor cocktail according to manufacturer’s recommendations (ThermoFisher). Whole-cell extracts for IRF3 dimerization detection were obtained using NativePAGE sample prep kit (ThermoFisher). Nuclear and cytoplasmic fractions were obtained using the NE-PER® nuclear and cytoplasmic extraction reagents (ThermoFisher) as per manufacturer’s instructions. Protein concentration was quantitated using the BCA protein assay kit (ThermoFisher).

### SDS-PAGE and immunoblotting

Denatured proteins were resolved using either 4–12% Bis-Tris gels or 10–20% tricine gels (ThermoFisher) according to manufacturer’s recommendations. Proteins resolved on Bis-Tris gels and tricine gels were transferred respectively onto 0.45 μm and 0.22 μm nitrocellulose membranes (ThermoFisher) and probed with appropriate primary antibodies (Additional file 1: Table S3). Secondary antibodies conjugated with IRDye® 800CW or 680RD (LI-COR Biosciences) were used and images captured with the Odyssey® infrared imaging system (LI-COR Biosciences). For densitometric quantification, the intensity of the bands was determined using Image Studio™ Lite software (LI-COR Biosciences).

### IRF3 native PAGE and immunoblotting

IRF3 dimerization assay was performed as described previously with minor modifications [14]. Mini-protean® TGX™ pre-cast gels (7.5%, Bio-Rad) were pre-run with the running buffer (25 mM Tris-HCl pH 8.4, 192 mM glycine in the presence or absence of 1% sodium deoxycholate in the cathode and anode buffer, respectively) at 40 mA for 30 min. Loading samples were prepared using NativePAGE™ sample prep kit (ThermoFisher) and applied to the gel for running. Proteins were then transferred onto a nitrocellulose membrane (ThermoFisher) and fixed with a solution containing 7% acetic acid, 40% ethanol and 3% glycerol in ddH2O. Membranes were then washed with 1X PBS and total protein stain was performed for normalization purposes using REVERT total protein stain according to manufacturer’s protocol (LI-COR Biosciences). Membranes were imaged using the 700 channel on the Odyssey® imaging system and processed for immunoblotting as indicated above.

### siRNA IRF3

Transient knockdown of *IRF3* in human primary AECs was achieved by transfection of specific siRNA (Dharmacon; Additional file 1: Table S4 for sequence of siRNAs). Briefly, cells were cultured in 12-well plates until reaching 75% confluency. The culture medium was then replaced with antibiotic-free medium BEGM and siRNAs were transfected using Lipofectamine® RNAiMAX (ThermoFisher) following manufacturer’s instructions. Briefly, 2 μl of siRNA (10 μM) was mixed with 1 μl of RNAiMAX and cells were transfected for 24h. Knockdown efficiency was validated by western blot. The culture medium was then replaced with steroid-free medium 24h prior to treatment with MEKi (10 nM for 1h) or DMSO control. Cells were then stimulated with poly(I:C) and sampled at the indicated time points. Whole cell lysis and RNA extraction was performed as indicated above.

### Cytokine quantification

Cell supernatants were collected at the indicated time points and secreted cytokines quantified by AlphaLISA (IFN-β; RANTES/CCL5; IL-1β; IL-6; IL-8/CXCL8;PerkinElmer) and ELISA (IFN-λ1; R&D Systems) according to the manufacturer’s instructions.

### Quantitative RT-PCR assay

Total RNA was extracted using RNeasy plus mini or RNeasy plus 96 kit (Qiagen) according to manufacturer’s instructions, and then reverse transcribed using random hexamers (ThermoFisher). Quantitative real-time PCR was performed using the TaqMan™ gene expression master mix (ThermoFisher) on QuantStudio™ 7 flex real-time PCR system (ThermoFisher). The sequences of primer/probe for qRT-PCR analysis are listed in the Additional file 1: Table S5. Fold induction was calculated using 2^-ΔΔCt^ normalized to the expression of β-actin and GAPDH genes.

### Quantification of viral RNA and live virus assay

RV2 or RSVA2 intracellular RNA was reverse transcribed using RV or RSV-specific primers and cDNA amplified using RV or RSV TaqMan® primer-probe mix and oasig™ onestep qRT-PCR mastermix (Primerdesign). Standard curves were generated using 10-fold serial dilutions of RV or RSV-positive control. Viral gene copy number was calculated by plotting cycle threshold on each standard curve. In order to quantify viral transcription in early infection, an oligo (dT) primer was used to reverse transcribe only mRNA (ThermoFisher). cDNA was then amplified using RV or RSV TaqMan® primer-probe mix and Precision® PLUS qPCR master mix (Primerdesign).

Infectious RV2 released into the supernatant was quantified using viral ToxGlo™ assay (Promega) according to manufacturer’s instructions. Briefly, H1-HeLa cells were cultured in 96-well plates and a serial dilution of supernatants containing the infectious virus was performed and applied to the cells until visual detection of cytopathic effects. The viral ToxGlo™ reagent was then added and luminescence was measured. The virus dilution that produced a cytotoxic endpoint effect (50% CPE) was then calculated.

### MEK1, ERK and AKT surefire® ultra™ assay

The Alpha SureFire® Ultra™ multiplex assay was used to simultaneously quantify phosphorylated (pAKT S473) and total AKT (tAKT), or phosphorylated (pERK1/2 T202/Y204) and total ERK1/2 as per manufacturer’s recommendations (PerkinElmer). Total MEK1 was quantified using SureFire® Ultra™ assay.

### LDH assay

Cytotoxicity was measured by detection of lactate dehydrogenase (LDH) released in the cell supernatant with the cytotoxicity assay kit according to the manufacturer’s instructions (Promega).

### Confocal microscopy

Cells were cultured in 96-well plates until 75% confluency, fixed for 10 min in 4% buffered formalin followed by three washes with PBS. After 10 min permeabilization in 0,2% triton-X-100 and three additional PBS washes cells were blocked for 1h in 5% BSA + 5% FBS in PBS. Following an incubation in primary antibody (anti-IRF3) diluted in blocking buffer and three PBS washes, cells were incubated for an additional 1h in secondary antibody (anti-rabbit, Alexa Fluor 594) + Hoechst33258 diluted in PBS. After three PBS washes, cells were covered with PBS and the plate was sealed with a black adhesive seal. Cells were imaged at optimised imaging conditions using a CellVoyager CV7000 (Yokogawa) spinning disk microscope imaging 20 × 5 fields of view per well. Images were processed using Fiji and nuclear translocation was quantified by calculating nuclear to cytoplasmic ratio, nuclei were segmented using the Hoechst channel (405/450) followed by calculating pixel intensities for IRF3 using the far-red channel (600/637). Cytoplasmic area was segmented using the far-red channel followed by calculating pixel intensities for IRF3 and the nuclear to cytoplasmic ratio was calculated and plotted.

### Statistical analysis

Analyses were performed using R 3.5.0 [15] and R Studio 1.1.453 [16]. Data was first imported and transformed using the readxl [17] and tidyverse [18] packages. Log2 transformations were applied to the fold changes between treatment and reference groups (untreated or virus alone). The qqplotr package [19] was used to create Q-Q plots with confidence intervals to visually inspect if the data were normally distributed. The non-parametric Wilcoxon matched pairs signed rank test was used to test the statistical significance of fold changes. A *p*-value less than 0.05 was considered statistically significant.

## Results

### MEKi reduces viral load in RV2 challenged AECs but not those challenged with RSVA2

**Fig. 1:**
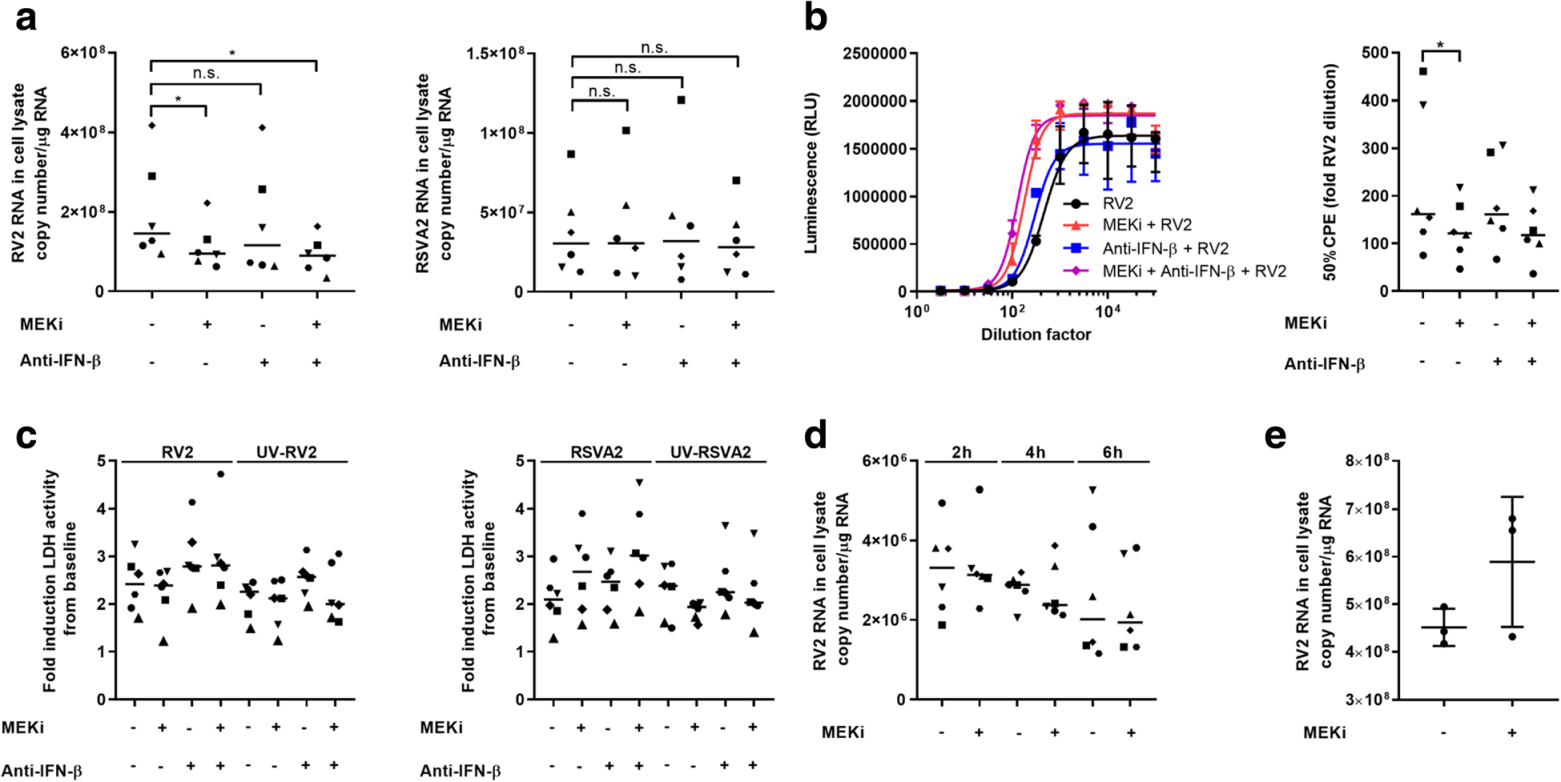
MEKi reduces viral load in RV2 challenged AECs but not those challenged with RSVA2. **a** Total RNA was extracted from AECs 24h p.i. (MOI 0.1) and then reverse-transcribed using RV or RSV-specific primers. RV2 or RSVA2 vRNA copy number was quantified by RT-qPCR. **b** RV2 live virus containing supernatants were collected at 24h p.i. and were used to infect permissive H1-HeLa cells. 50% CPE was determined using viral ToxGlo™ kit. **c** Cytotoxicity analysis of LDH release in the supernatants of AECs pretreated with DMSO, MEKi, or anti-IFN-β antibody for 1h followed by infection with or without RV2 or RSVA2 or UV-inactivated viruses for 24h (MOI 0.1). **d** Total RNA was extracted from AECs infected with RV2 for 2-6h (MOI 0.1) and mRNA was reverse transcribed using oligo (dT) primers. RV2 copy number was quantified by RT-qPCR. **e** RV2 copy number was quantified by RT-qPCR using total RNA extracted from H1-HeLa cells 24h p.i. (MOI 0.1). Each symbol () represents a donor and the horizontal bars represent the grand median in **a**, **c**, and **d** (*n* = 6). Data are presented as median and error bars represent 95% confidence interval (*n* = 6). Three independent experiments in **e**. Statistical analysis was performed with the Wilcoxon signed-rank test. *p < 0.05 indicates statistical significance

The impact of inhibition of MEK on viral load was assessed through the measure of total intracellular viral RNA (Fig. 1a). Based on the potential crosstalk between MEK/IFN signaling [12] and the importance of IFN-β in the protection of AECs against RV [20], the concentration of the MEK inhibitor (MEKi), 10 nM, was predetermined by dose response in relation to IFN-β production (EC50 = 3 nM; Additional file 1: Figure S1a). MEKi significantly reduced RV2 infection by nearly 40% (Fig. 1a) in presence or absence of a neutralizing antibody against IFN-β (anti-IFN-β). On the other hand, treatment with MEKi did not result into a decrease of RSVA2 load (Fig. 1a), highlighting the ability of RSVA2 to negate the positive effects of MEKi. Target engagement was confirmed by suppression of pERK1/2 (Additional file 1: Figure S1b), and this also showed that neither RV2 nor RSVA2 modulate MEK pathway within 24h.

To confirm the effect of MEKi on RV2 load, we determined the live virus burden in supernatants using the viral ToxGlo™ kit (Fig. 1b) [21]. In agreement with Fig. 1a, MEKi significantly reduced the cytopathic effect (CPE) by 34% in RV2-infected H1-HeLa cells (*p* < 0.05) as determined by the 50% CPE measurement. The release of lactate dehydrogenase (LDH), a marker of cytotoxicity, by virus-infected cells was not substantially modulated by MEKi indicating that MEKi did not impair cell viability in the effective concentrations used (Fig. 1c and Additional file 1: Figure S1c).

To rule out any possible effect of MEKi on viral entry and penetration, we quantified RV2 transcription in early infection using oligo (dT) primers as RV2 RNA genome is poly(A)-tailed [22] (Fig. 1d). Viral transcription followed a similar kinetic in AECs treated with MEKi compared to controls, suggesting no difference in the amount of viral genomic template available for transcription early in infection.

To assess whether MEKi has a direct effect on the life cycle of RV2 in an IFN-independent manner, we infected H1-HeLa cells that replicate features of RV infection in primary AECs but don’t produce IFNs [23, 24] (Fig. 1e). There was no induction of *IFNB1* or *IFNL1* mRNA (Additional file 1: Figure S1d) and this was associated with an absence in the reduction of RV2 load in H1-HeLa, suggesting that MEK pathway is unlikely to be directly hijacked by RV2 for replication purposes.

### MEKi enhances type I IFN response following RV2 or RSVA2 infection

**Fig. 2:**
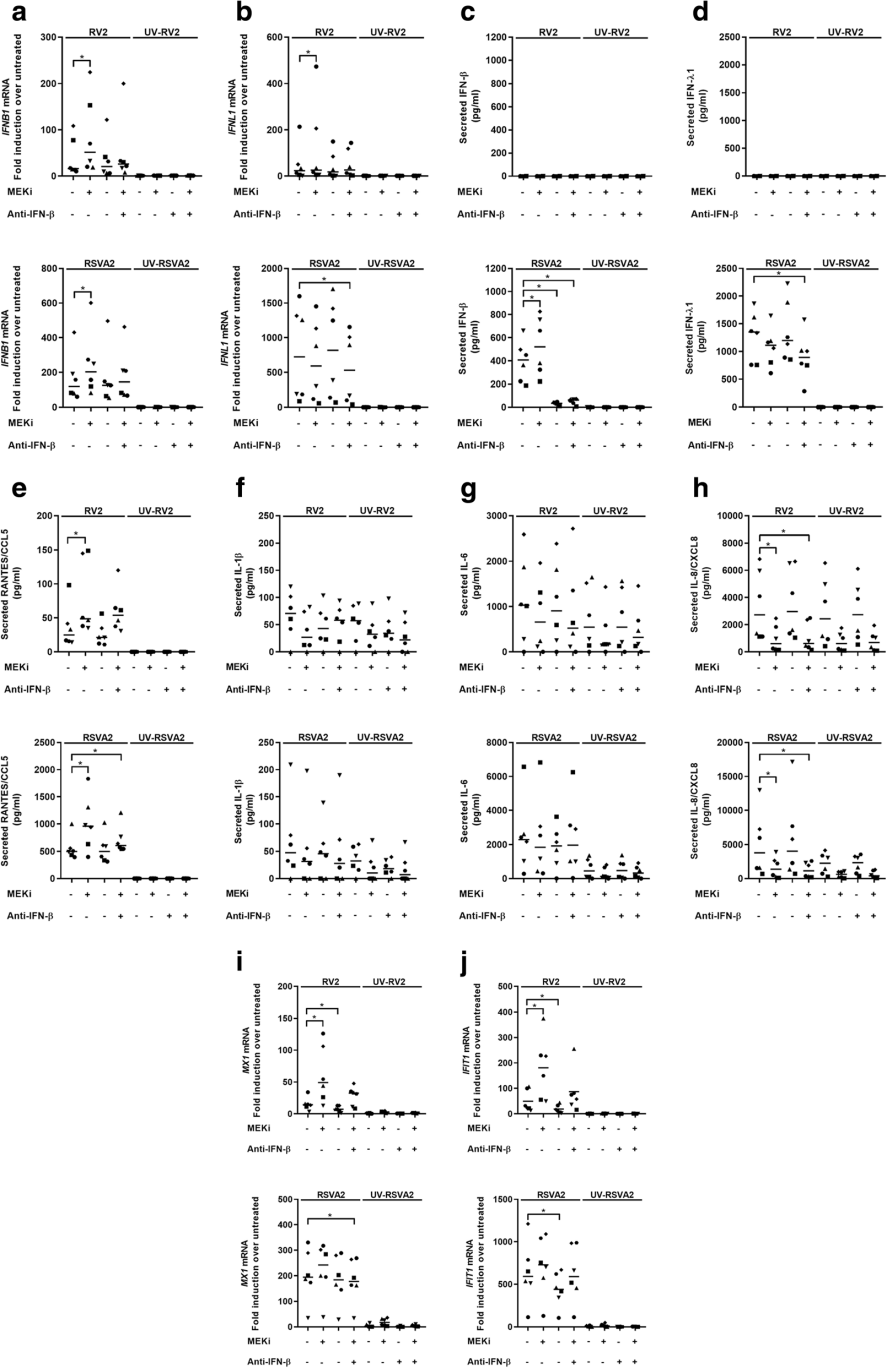
MEKi enhances type I IFN response following RV2 or RSVA2 infection. **a** qRT-PCR analysis of *IFNB1* mRNA in AECs pretreated with DMSO, MEKi, or anti-IFN-β antibody for 1h followed by infection with or without RV2 or RSVA2 or UV-inactivated viruses for 24h (MOI 0.1). **b** qRT-PCR analysis of *IFNL1* mRNA in AECs pretreated with DMSO, MEKi, or anti-IFN-β antibody for 1h followed by infection with or without RV2 or RSVA2 or UV-inactivated viruses for 24h (MOI 0.1). **c** AlphaLISA analysis of IFN-β in the supernatants of AECs pretreated with DMSO, MEKi, or anti-IFN-β antibody for 1h followed by infection with or without RV2 or RSVA2 or UV-inactivated viruses for 24h (MOI 0.1). **d** ELISA analysis of IFN-λ1 in the supernatants of AECs pretreated with DMSO, MEKi, or anti-IFN-β antibody for 1h followed by infection with or without RV2 or RSVA2 or UV-inactivated viruses for 24h (MOI 0.1). **e** AlphaLISA analysis of RANTES/CCL5 in the supernatants of AECs pretreated with DMSO, MEKi, or anti-IFN-β antibody for 1h followed by infection with or without RV2 or RSVA2 or UV-inactivated viruses for 24h (MOI 0.1). **f** AlphaLISA analysis of IL-1β in the supernatants of AECs pretreated with DMSO, MEKi, or anti-IFN-β antibody for 1h followed by infection with or without RV2 or RSVA2 or UV-inactivated viruses for 24h (MOI 0.1). **g** AlphaLISA analysis of IL-6 in the supernatants of AECs pretreated with DMSO, MEKi, or anti-IFN-β antibody for 1h followed by infection with or without RV2 or RSVA2 or UV-inactivated viruses for 24h (MOI 0.1). **h** AlphaLISA analysis of IL-8/CXCL8 in the supernatants of AECs pretreated with DMSO, MEKi, or anti-IFN-β antibody for 1h followed by infection with or without RV2 or RSVA2 or UV-inactivated viruses for 24h (MOI 0.1). **i** qRT-PCR analysis of *MX1* mRNA in AECs pretreated with DMSO, MEKi, or anti-IFN-β antibody for 1h followed by infection with or without RV2 or RSVA2 or UV-inactivated viruses for 24h (MOI 0.1). **j** qRT-PCR analysis of *IFIT1* mRNA in AECs pretreated with DMSO, MEKi, or anti-IFN-β antibody for 1h followed by infection with or without RV2 or RSVA2 or UV-inactivated viruses for 24h (MOI 0.1). Each symbol () represents a donor and the horizontal bars represent the grand median in **a**-**j** (*n* = 6). Statistical analysis was performed with the Wilcoxon signed-rank test. *p < 0.05 indicates statistical significance

To determine the role of MEK1/2 signaling in relation to IFN production in primary AECs, IFN response was determined following infection with RV2 or RSVA2 in presence or absence MEKi (Fig. 2).

Both RV2 and RSVA2 induce *IFNB1* mRNA (encoding IFN-β) and this response was further amplified in presence of MEKi (2-fold, *p* < 0.05, 1.4-fold, *p* < 0.05, respectively) (Fig. 2a). Treatment with a neutralizing antibody against IFN-β offset the improved response caused by MEKi as comparable levels of *IFNB1* mRNA were induced compared to virus alone. We then investigated the expression of type III IFN (IFN-λ1), which revealed viral differences. MEKi enhanced expression of *IFNL1* mRNA in response to RV2 compared to RV2 alone (1.8-fold, *p* < 0.05) but not in response RSVA2, which in contrast was associated with a reduction (0.8-fold) (Fig. 2b).

IFN-β (Fig. 2c) and IFN-λ (Fig. 2d) productions were detected in response to RSVA2, but not to RV2. MEKi induced a higher secretion of IFN-β by RSVA2-infected cells compared to RSVA2 alone (1.3-fold, *p* < 0.05) (Fig. 2c). In contrast, there was a reduction in the production of RSVA2-induced IFN-λ1 in the presence of MEKi (0.8-fold, *p* = 0.0625) compared to RSVA2 alone (Fig. 2d), indicating that type I and III IFN responses are differentially regulated by RSVA2. In addition, MEKi alone did not induce any IFN response, suggesting that inhibition of MEK pathway does not directly activate IFN system and requires TLR3/RIG-I activation.

Off-target effects of small molecule inhibitors are a common problem when interpreting their biological effects. Therefore, we performed additional experiments with two MEK inhibitors, which are both structurally unrelated to MEKi (Additional file 1: Figure S2a and Table S2). We could observe the same effect of MEK inhibition on secreted IFN-β levels, confirming that the enhanced release of IFN-β upon TLR3 stimulation is not an off-target effect of MEKi. To further rule out the effect of MEKi to be off target, we used one of our in-house designed, synthesized and validated MEK proteolysis targeting chimeras (PROTACs) to study the effect of MEK inhibition by protein degradation for the release of IFN-β upon poly(I:C) stimulation of AECs (Vollmer, in preparation). Under the conditions of MEK degradation and suppression of ERK1/2 phosphorylation (Additional file 1: Figure S2b), we observed an enhanced IFN-β secretion by the MEK1 PROTAC and the respective parental MEK1 small molecule inhibitor compared to the poly(I:C) alone treatment (Additional file 1: Figure S2b) (2-fold increase, *p* < 0.05).

To determine whether the effect of MEKi had an impact on the inflammatory response, we measured secretion of RANTES/CCL5 (Fig. 2e), which is also a target of IRF3 [25]. In agreement with the IFN data (Fig. 2c), MEKi boosted the production of RANTES/CCL5 in response to RV2 (2.4-fold, *p* < 0.05) or RSVA2 (1.6-fold, *p* < 0.05) compared to their respective controls. We also quantified the expression of NF-κB pro-inflammatory cytokines including IL-1β (Fig. 2f), IL-6 (Fig. 2g), and IL-8/CXCL8 (Fig. 2h). MEKi did not modulate production of IL-1β (Fig. 2f) or IL-6 (Fig. 2g) following RV2 or RSVA2 infection. However, it caused a 5.3 and 3.2-fold reduction in IL-8/CXCL8 production (Fig. 2h) by RV2 and RSVA2-infected cells, respectively (*p* < 0.05). Taken together, these data suggest that enhanced IFN response is not driven by aberrant inflammation. Importantly, it demonstrates that MEKi has a dual ability to reduce the inflammatory response (IL-8/CXCL8) and boost the antiviral response (IFNs and RANTES/CCL5).

The influence of increased IFN-β production on canonical ISGs, MX1 (Fig. 2i) and IFIT1 (Fig. 2j), also revealed viral differences as MEKi further enhanced expression of RV2-induced *MX1* and *IFIT1* mRNA compared to RV2 alone (3.6-fold, *p* < 0.05,3.7-fold, *p* < 0.05, respectively) while there was no enhancement effect following RSVA2 infection (Fig. 2i and j).

To further highlight viral differences, we used poly(I:C) as a control, since it induces IFN-β production through TLR3/IRF3 signaling [26]. After confirmation of target engagement (Additional file 1: Figure S2c), IFN and the inflammatory response was characterised as previously described (Additional file 1: Figure S2d-f). Essentially, poly(I:C) control recapitulated the outcome observed with RV2 challenge where MEKi significantly induced a higher expression of type I and III IFNs (mRNA and protein levels) (Additional file 1: Figure S2d and e), ISGs (*MX1* and *IFIT1* mRNA) (Additional file 1: Figure S2f), and RANTES/CCL5 production (Additional file 1: Figure S2e) compared to poly(I:C) alone. Taken together, these data show that disruption of MEK signaling affects both the type I and the type III IFN response, with the latter more sensitive to viral regulation, particularly by RSVA2.

### MEKi does not modulate TBK1 activation but upregulates activity of AKT

**Fig. 3:**
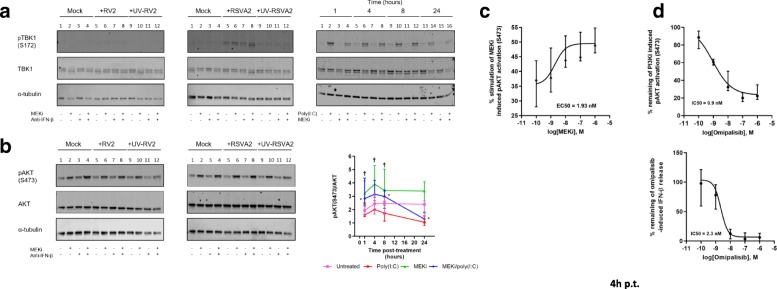
MEKi does not modulate TBK1 activation but upregulates activity of AKT. **a** Immunoblot analysis (with anti-pTBK1, anti-TBK1, and anti-α-tubulin) of AECs pretreated with DMSO, MEKi, or anti-IFN-β antibody for 1h followed by infection with or without RV2 or RSVA2 or UV inactivated viruses for 24h (MOI 0.1). For the poly (I:C) model (right panel), immunoblot analysis of AECs pretreated with DMSO or MEKi for 1h and subsequently stimulated with or without poly(I:C) for 1-24h. **b** Immunoblot analysis (with anti-pAKT, anti-AKT, and anti-α-tubulin) of AECs pretreated with DMSO, MEKi, or anti-IFN-β antibody for 1h followed by infection with or without RV2 or RSVA2 or UV inactivated viruses for 24h (MOI 0.1). For the poly(I:C) model (right panel), simultaneous quantification of pAKT / total AKT was determined in AECs pretreated with DMSO or MEKi for 1h and subsequently stimulated with or without poly(I:C) for 1-24h. **c** Simultaneous quantification of pAKT / total AKT in AECs pretreated with MEKi at the indicated concentration and EC50 determined accordingly. **d** Simultaneous quantification of pAKT / total AKT in AECs pretreated with PI3Ki at the indicated concentration and IC50 determined accordingly. AlphaLISA analysis of IFN-β in the supernatants of AECs pretreated PI3Ki for 1h followed by stimulation with poly (I:C) for 4h. † relative to MEKi vs untreated and * relative to MEKi/poly(I:C) vs untreated. **a** and **b** are representative immunoblots (*n* = 6). Data are presented as median ± interquartile range in b (poly (I:C) model), **c** and **d**. (*n* = 6). Statistical analysis was performed with the Wilcoxon signed-rank test. * or † *p* < 0.05 indicates statistical significance

To determine whether TLR3/RIG-I signaling was altered in the presence of MEKi, we measured the activation of TBK1 (pTBK1 S172), an upstream activator of IRF3 (Fig. 3a). MEKi did not modulate activation of TBK1 following RSVA2 infection compared to RSVA2 alone (Fig. 3a, middle panel, lane 6 vs lane 5; Additional file 1: Figure S3a). A similar response was observed with RV2 challenge although the signal was much weaker.

Poly(I:C) induced a strong and rapid activation of TBK1, visible after 1h, and there was no modulation due to MEKi (Fig. 3a, right panel; Additional file 1: Figure S3a). This suggests that activation of TBK1 occurs normally in stimulated cells treated with MEKi.

We next examined the activation of AKT (pAKT S473) (Fig. 3b), a positive regulator of the IFN response, which could also be induced via a feedback mechanism following MEK inhibition [27]. MEKi alone induced a higher activation of AKT compared to controls by 24h p.i. (Fig. 3b, lane 2 vs lane 1, 2-fold, *p* < 0.05; Additional file 1: Figure S3b). However, neither RSVA2 nor RV2 modulated pAKT as levels were comparable to untreated cells (Fig. 3b, lane 5 vs lane 1). In addition, the enhanced activation of AKT due to MEKi was also observed in cells infected with RV2 or RSVA2, further confirming that both viruses do not modulate AKT activation by 24h p.i. (Fig. 3b, lane 6 vs lane 5, 2-fold, *p* < 0.05). In response to poly(I:C), there was a slow and steady decline of AKT activation compared to untreated cells (Fig. 3b, right panel). Remarkably, MEKi induced a hyperactivation of AKT as early as 1h p.t and a further increase by 4h p.t. compared to untreated cells (1.9-fold, *p* < 0.05, 1.6-fold, *p* < 0.05, respectively). A decrease was then observed from 8 to 24h p.t., although the degree of activation remained higher compared to untreated cells (1.5-fold, *p* < 0.05, 1.3-fold, *p* = 0.0625, respectively). In response to MEKi/poly(I:C), the kinetic of AKT activation followed the pattern observed with cells treated with MEKi alone except that the slope on the second phase of AKT activation kinetic was steeper compared to MEKi-treated cells (sharp reduction). To confirm the effect of MEKi on pAKT, we performed a dose response on cells treated with MEKi alone for 4h and the results clearly demonstrate that MEKi induced AKT phosphorylation at S473 in a dose-dependent manner with an EC50 value of approximately 2 nM (Fig. 3c). To further validate the involvement of PI3K/AKT pathway, we stimulated AECs with poly(I:C) in the presence or absence of a pharmacological inhibitor of PI3K (Omipalisib, PI3Ki), which is the upstream kinase that activates AKT (Fig. 3d). PI3Ki not only inhibited AKT activation but also reduced IFN-β secretion in a dose-dependent manner (Fig. 3d) without causing cytotoxicity (Additional file 1: Figure S3c and d). Consistent with the literature, these data demonstrate an essential role of PI3K/AKT in mounting IFN-β response.

### MEKi does not increase IRF3 activation nor its translocation into the nucleus

**Fig. 4:**
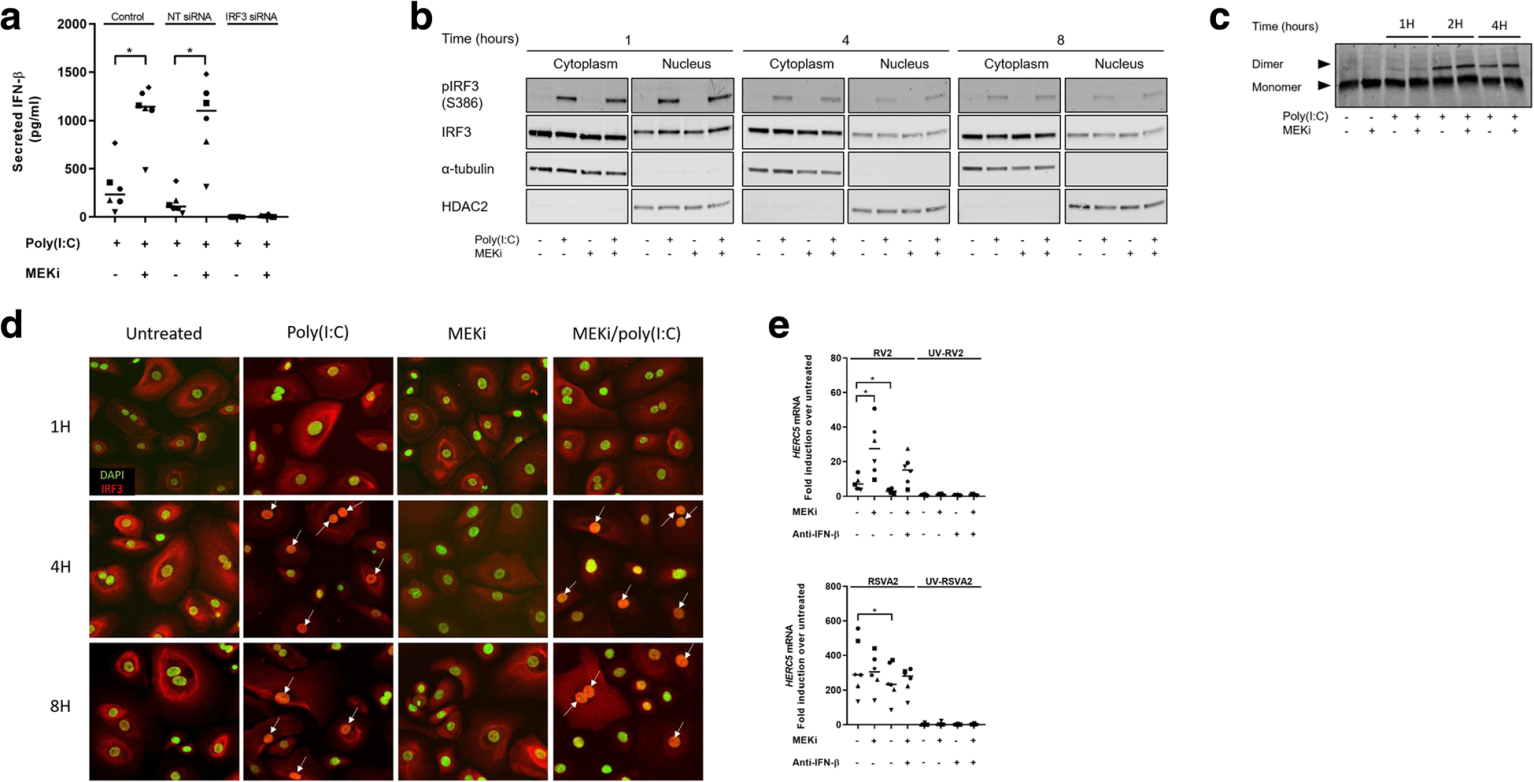
MEKi does not increase IRF3 activation nor its translocation into the nucleus. **a** AlphaLISA analysis of IFN-β in the supernatants of AECs transfected with IRF3 or NT siRNA followed by treatment with DMSO or MEKi and subsequent stimulation with poly(I:C) for 4h. **b** Immunoblot analysis (with anti-pIRF3, anti-IRF3, α-tubulin, and HDAC2) of fractionated AECs pretreated with DMSO or MEKi for 1h followed by stimulation with or without poly(I:C) for 1-8h. **c** Native immunoblot analysis (with anti-IRF3) of AECs pretreated with DMSO or MEKi for 1h followed by stimulation with or without poly(I:C) for 1-4h. **d** Confocal microscopy analysis of IRF3 translocation in AECs pretreated with DMSO or MEKi for 1h followed by stimulation with or without poly(I:C) for 1-8h. **e** qRT-PCR analysis of *HERC5* mRNA in AECs pretreated with DMSO, MEKi, or anti-IFN-β antibody for 1h followed by infection with or without RV2 or RSVA2 or UV-inactivated viruses for 24h (MOI 0.1). Each symbol () represents a donor and the horizontal bars represent the grand median in **a** and **e** (*n* = 6). **b** and **c** are representative immunoblots (*n* = 6). **d** is representative of confocal images (*n* = 6). Statistical analysis was performed with the Wilcoxon signed-rank test. * *p* < 0.05 indicates statistical significance

IRF3 is a critical transcription factor that is constitutively expressed in AECs, thus enabling to quickly respond to viral infection. Knockdown of IRF3 expression by small interfering RNA (siRNA) (Additional file 1: Figure S4a and b) abolished IFN-β secretion confirming its utmost importance in mounting an early IFN response (Fig. 4a). We used the poly(I:C) model to study key hallmarks of IRF3 activation, phosphorylation and dimerization, as these were not detectable at 24h p.i. in response to RV2 or RSVA2 (Additional file 1: Figure S4c). We first measured phosphorylated IRF3 (pIRF3 S386) in cytoplasmic and nuclear fractions. There was a rapid phosphorylation of IRF3 (1h p.t.) in response to poly(I:C) and MEKi did not affect the ratio of pIRF3 over total IRF3 in stimulated cells as the levels were similar to those in response to poly(I:C) alone (Fig. 4b and Additional file 1: Figure S4d). We then examined IRF3 dimerization by native gel and we found concordantly that MEKi did not change the level of IRF3 dimer in presence of poly(I:C) (Fig. 4c and Additional file 1: Figure S4e). To corroborate these findings, we investigated the kinetic of IRF3 nuclear translocation by confocal immunofluorescence (Fig. 4d and Additional file 1: Figure S4f). The translocation of IRF3 peaked at 4h p.t. with no apparent difference between the two treatments, suggesting that MEKi does not modulate IRF3 translocation. Taken together, these findings suggest that MEKi enhances IFN-β production through IRF3 signaling possibly without interfering with IRF3 activation process. Moreover, increased AKT activation due to MEKi clearly does not affect IRF3 activation, suggesting other AKT-dependent events contribute to enhanced type I IFN response in AECs.

We examined the expression of an ISG, HERC5, which is a ligase that mediates ISGylation of IRF3, thus promoting subsequently its activity [28] (Fig. 4e). Both viruses induced expression of *HERC5* mRNA, particularly RSVA2 which showed a greater potency compared to RV2 (54-fold difference). In addition, there was another noticeable difference between the viruses, as MEKi further augmented the expression of *HERC5* mRNA compared to RV2 alone (3.6-fold, *p* < 0.05), whereas RSVA2 induction of *HERC5* mRNA was not impacted by MEKi. In agreement with the previous observations, MEKi/poly(I:C) significantly induced higher level of *HERC5* mRNA by 24h compared to poly(I:C) (2-fold, *p* < 0.05) (Additional file 1: Figure S4g).

In addition to IRF3, other IRFs have been involved in the amplification of type I IFN response following viral infection: IRF7 plays an instrumental role [3] whereas IRF1 and IRF5, are reported to be dispensable for inducing type I IFN [29, 30]. We therefore sought to determine whether MEKi had an effect of IRF1, 5 and − 7 expression (Additional file 1: Figure S4h-j). In accordance with previous results pertaining to ISGs, MEKi enhanced the expression of *IRF1* and *IRF7* mRNA in RV2-infected cells compared to RV2 alone (3.2-fold, *p* < 0.05, 2.5-fold, *p* < 0.05, respectively) (Additional file 1: Figure S4h and j). Although RSVA2 significantly induced expression of *IRF1* and *IRF7* mRNA, there was no positive effect of MEKi. Poly(I:C) control also showed that MEKi boosted expression of *IRF1* (1.5-fold at 4h, *p* < 0.05, 1.6-fold at 8h, *p* < 0.05, 2.2-fold at 24h, *p* < 0.05, respectively) and *IRF7* mRNA (1.3-fold, *p* = 0.094), although statistical significance was not reached for the latter (Additional file 1: Figure S4h and j). Nuclear translocation of IRF7 in human plasmacytoid dendritic cells (pDCs) has been shown to be PI3K-AKT dependent [31], and we performed a translocation study and assessed whether MEKi-induced increased AKT activation has an impact on IRF7 translocation. MEK inhibition did not result into elevated IRF7 nuclear translocation but was rather associated with decreased expression of cytoplasmic IRF7 (0.7-fold at 24h, *p* < 0.05) (Additional file 1: Figure S4k), suggesting that the second wave of IFN response attributable to newly synthesized IRF7 is unlikely to contribute to enhanced type I and III IFN production. It rather indicates that early events causing enhanced IFN production at an early stage have an incidence on IRF7 expression at a later stage. *IRF5* mRNA was poorly induced in stimulated cells, suggesting that IRF5 does not participate in the IFN response in the airway epithelium (Additional file 1: Figure S4i).

### MEKi does not affect IFNAR signaling and its negative feedback loop

**Fig. 5:**
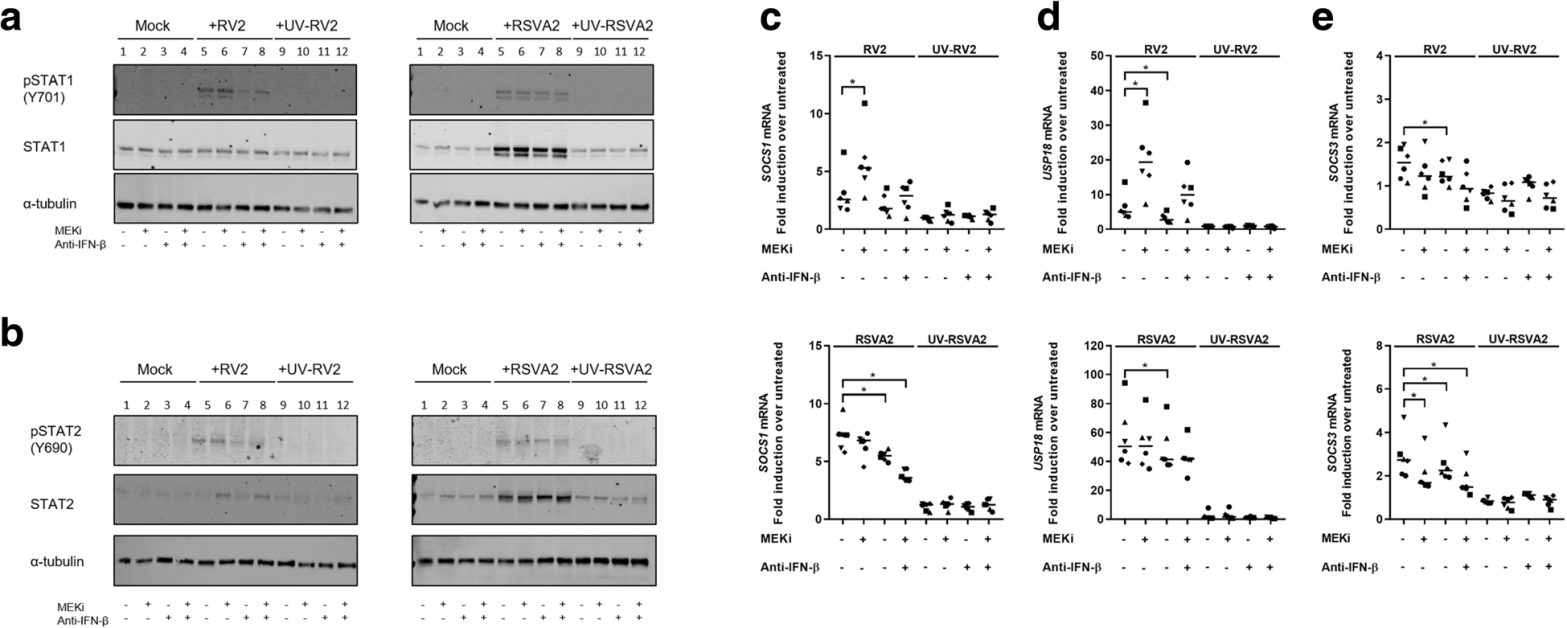
MEKi does not affect IFNAR signaling and its negative feedback loop. **a** Immunoblot analysis (with anti-pSTAT1, anti-STAT1 and anti-α-tubulin) of AECs pretreated with DMSO, MEKi, or anti-IFN-β antibody for 1h followed by infection with or without RV2 or RSVA2 or UV-inactivated viruses for 24h (MOI 0.1). **b** Immunoblot analysis (with anti-pSTAT2, anti-STAT2 and anti-α-tubulin) of AECs pretreated with DMSO, MEKi, or anti-IFN-β antibody for 1h followed by infection with or without RV2 or RSVA2 or UV-inactivated viruses for 24h (MOI 0.1). **c** qRT-PCR analysis of *SOCS1* mRNA in AECs pretreated with DMSO, MEKi, or anti-IFN-β antibody for 1h followed by infection with or without RV2 or RSVA2 or UV-inactivated viruses for 24h (MOI 0.1). **d** qRT-PCR analysis of *USP18* mRNA in AECs pretreated DMSO, MEKi, or anti-IFN-β antibody for 1h followed by infection with or without RV2 or RSVA2 or UV-inactivated viruses for 24h (MOI 0.1). **e** qRT-PCR analysis of *SOCS3* mRNA in AECs pretreated with DMSO, MEKi, or anti-IFN-β antibody for 1h followed by infection with or without RV2 or RSVA2 or UV-inactivated viruses for 24h (MOI 0.1). **a** and **b** are representative immunoblots (*n* = 6). Each symbol () represents a donor and the horizontal bars represent the grand median in **c**, **d**, **e** (*n* = 6). Statistical analysis was performed with the Wilcoxon signed-rank test. * *p* < 0.05 indicates statistical significance

To determine if MEKi-induced enhanced IFN production is partly due to increased IFNAR signaling, we quantified the expression of STAT1 and STAT2, which are also ISGs, and their activation (pSTAT1 Y701; pSTAT2 Y689) (Fig. 5a and b).

RV2 did not induce higher expression of STAT1 compared to untreated cells, as opposed to RSVA2 which significantly increased expression of STAT1 (lane 5 vs lane 1, 4.7-fold, *p* < 0.05), thus correlating with its superior potency in activating the IFN response (Fig. 5a and Additional file 1: Figure S5a). However, MEKi did not increase the expression of STAT1 following RSVA2 infection (lane 6 vs lane 5), which is line with other induced ISGs. As for the activation STAT1, RV2 or RSVA2 both induced phosphorylation of STAT1 but there was no significant modulation due to MEKi (Fig. 5a, lane 6 vs lane 5; Additional file 1: Figure S5a). The presence of the antibody against IFN-β disrupted the activation of STAT1 (Fig. 5a, lane 7 vs lane 5; Additional file 1: Figure S5a), particularly following RV2 infection (lane 7 vs lane 5, 0.7-fold, *p* < 0.05; Additional file 1: Figure S5a).

RV2 was a poor inducer of STAT2 compared to RSVA2, the latter significantly increasing STAT2 expression compared to controls (Fig. 5b, lane 5 vs lane 1, 5.7-fold, *p* < 0.05; Additional file 1: Figure S5b). However, there was a slight increase of expression of STAT2 in cells treated with MEKi and subsequently infected with RV2 (Fig. 5b, lane 6 vs lane 5, 1.7-fold, *p* < 0.05; Additional file 1: Figure S5b), which is in line with the effects observed on other ISGs. In contrast, there was no increase of STAT2 expression due to MEKi in response to RSVA2 infection (Fig. 5b, lane 6 vs lane 5; Additional file 1: Figure S5b), which indicates again that RSVA2 effectively disrupts IFN signaling. Similarly to the activation of pSTAT1, there was no effect of MEKi on pSTAT2 (Fig. 5b, lane 6 vs lane 5; Additional file 1: Figure S5b).

To further verify that MEKi did not have effects on the control of IFNAR signaling, we quantified the expression of negative regulators of IFNAR signaling including ubiquitin specific peptidase 18 (USP18) and suppressor of cytokine signaling (SOCS)-1 and  -3 [11]. In presence of MEKi, RV2 induced a higher expression of *SOCS1* and *USP18* mRNA compared to RV2 alone (2-fold, *p* < 0.05, 3.2-fold, *p* < 0.05, respectively) (Fig. 5c and d), which was associated with higher expression of *IFNB1* mRNA (Fig. 2a). In contrast, there was a trend towards a decreased expression of *SOCS1* mRNA expression following RSVA2 infection in cells treated with MEKi (Fig. 5c), and this despite an elevated IFN response in these cells compared to RSVA2 alone (Fig. 2a and c). Strikingly, there was a synergistic effect between RSVA2 and the neutralizing antibody against IFN-β in the reduction of both *SOCS1* and *USP18* mRNA expression compared to RSVA2 alone (0.8-fold, *p* < 0.05, 0.8-fold, *p* < 0.05, respectively) (Fig. 5c and d).

RV2 poorly induced the expression of *SOCS3* mRNA and similarly to *SOCS1* mRNA data, RSVA2 did cause a significant decrease of *SOCS3* mRNA expression in the presence of MEKi compared to RSVA2 alone (0.8-fold, *p* < 0.05) and this effect was further amplified with anti-IFN-β (0.7-fold, *p* < 0.05).

Interestingly, poly(I:C) model in relation to IFNAR signaling recapitulated features of both RV2 and RSVA2 infections. In presence of poly(I:C), MEKi upregulated expression of STAT1 compared to poly(I:C) alone (Additional file 1: Figure S5c, lane 16 vs lane 14, 1.7-fold, *p* < 0.05) whereas STAT2 was moderately induced (Additional file 1: Figure S5d). There was a steady expression of *SOCS1* mRNA over time and there was no modulation due to MEKi (Additional file 1: Figure S5e). In presence of poly(I:C), MEKi did induce a higher expression of *USP18* mRNA after 8h compared to poly(I:C) alone (1.3-fold at 8h, *p* < 0.05, 1.5-fold at 24 h, *p* = 0.0625) (Additional file 1: Figure S5f). Poly(I:C) induced a biphasic expression of *SOCS3* mRNA and MEKi/poly(I:C) followed a similar pattern except at 24h p.i. where there was a higher expression of *SOCS3* mRNA compared to poly(I:C) alone (1.4-fold, *p* < 0.05) (Additional file 1: Figure S5g). Taken together, these data suggest that MEKi does not adversely affect IFNAR signaling as higher expression of IFN-β due to MEKi was associated with increased expression of SOCS1 and USP18. However, it also shows that RSVA2 actively modulates these regulators.

### MEKi promotes deactivation (phosphorylation) of translational repressor 4E-BP1 but activates p70S6K

Translation control is a key mechanism that regulates IFN response and the PI3K-AKT-mTOR pathway has been shown to promote translation of ISGs, by inducing phosphorylation/inactivation of the eukaryotic translation initiation factor 4E binding protein1 (4E-BP1), in response to IFN treatment or TLR3 stimulation [7, 32, 33]. Hypophosphorylated form of 4E-BP1 binds and sequesters eIF4E, a limiting component of the eIF4F translation initiation complex, which ultimately represses the initiation of mRNA translation. To determine whether activity of 4E-BP1 was altered by MEKi, we quantified the expression of phospho-forms of 4E-BP1 named α (hypophosphorylated), β, and γ (hyperphosphorylated) using tricine gels optimized for high resolution of low molecular peptides [34].

**Fig. 6:**
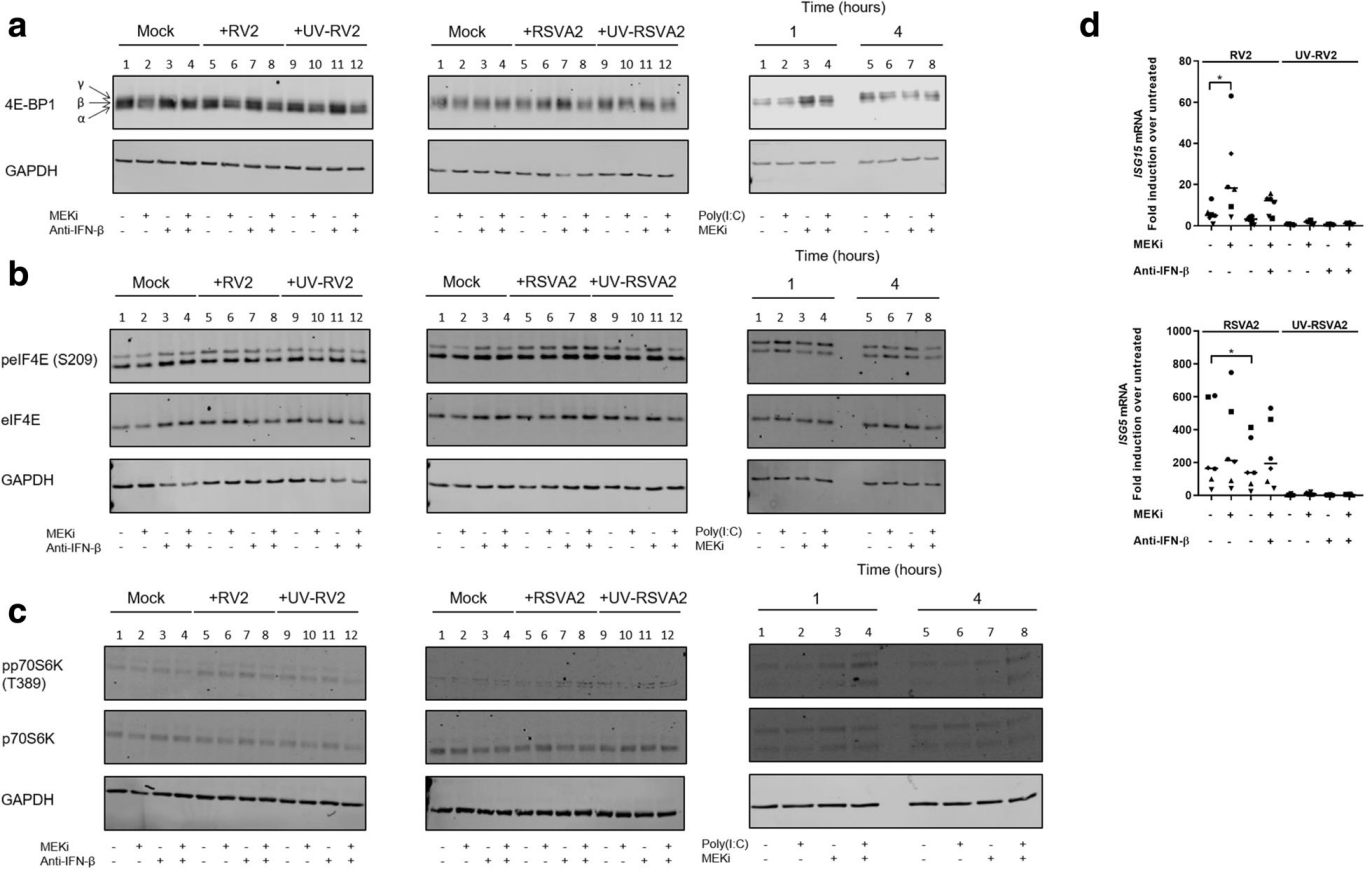
MEKi promotes deactivation (phosphorylation) of translational repressor 4E-BP1 but activates p70S6K. **a** Immunoblot analysis (with anti-4E-BP1 and anti-GAPDH) of AECs pretreated with DMSO, MEKi, or anti-IFN-β antibody for 1h followed by infection with or without RV2 or RSVA2 or UV-inactivated viruses for 24h (MOI 0.1). For the poly(I:C) model (right panel), immunoblot analysis of AECs pretreated with DMSO or MEKi for 1h and subsequently stimulated with or without poly(I:C) for 1-4h. Arrows show the α, β, and γ forms of 4E-BP1. α, β, and γ in order of increasing electrophoretic mobility, where γ form is the hyperphosphorylated form of 4E-BP1. β is phosphorylated to a lesser extent that γ, and α is the hypophosphorylated form. **b** Immunoblot analysis (with anti-peIF4E, anti-eIF4E and anti-GAPDH) of AECs pretreated with DMSO, MEKi, or anti-IFN-β antibody for 1h followed by infection with or without RV2 or RSVA2 or UV-inactivated viruses for 24h (MOI 0.1). For the poly(I:C) model (right panel), immunoblot analysis of AECs pretreated with DMSO or MEKi for 1h and subsequently stimulated with or without poly(I:C) for 1-4h. **c** Immunoblot analysis (with anti-pp70S6K, anti-p70S6K, and anti-GAPDH) of AECs pretreated DMSO, MEKi, or anti-IFN-β antibody for 1h followed by infection with or without RV2 or RSVA2 or UV-inactivated viruses for 24h (MOI 0.1). For the poly(I:C) model (right panel), immunoblot analysis of AECs pretreated with DMSO or MEKi for 1h and subsequently stimulated with or without poly(I:C) for 1-4h. **d** qRT-PCR analysis of *ISG15* mRNA in AECs pretreated with DMSO, MEKi, or anti-IFN-β antibody for 1h followed by infection with or without RV2 or RSVA2 or UV-inactivated viruses for 24h (MOI 0.1). **a**, **b**, and **c** are representative immunoblots (*n* = 6). Each symbol () represents a donor and the horizontal bars represent the grand median in **d** (*n* = 6). Statistical analysis was performed with the Wilcoxon signed-rank test. * *p* < 0.05 indicates statistical significance

Neither RV2 nor RSVA2 alone modulated phosphorylation/inactivation of 4E-BP1 by 24h compared to untreated controls and there was no effect of MEKi either at 24h p.i. (Fig. 6a and Additional file 1: Figure S6a). However, the time course experiment used in the poly(I:C) model shed more light in the phosphorylation/inactivation kinetic of 4E-BP1 (Fig. 6a, right panel; Additional file 1: Figure S6a). By 1h, MEKi induced phosphorylation/inactivation of 4E-BP1 compared to untreated control (Fig. 6a, right panel, lane 3 vs lane 1, 1.8-fold, *p* < 0.05; Additional file 1: Figure S6a) but this effect was not sustained beyond 1h, suggesting that phosphorylation/inactivation of 4E-BP1 is an early event that does not require IFN (Fig. 6a; Additional file 1: Figure S6a).

In addition to the inactivation of 4E-BP1 by the PI3K-AKT pathway, phosphorylation of eIF4E at serine 209 (S209) by ERK1/2-induced MAPK-interacting kinase (MNK) 1 (downstream target of p38 and ERK1/2) has been proposed as an additional mechanism for promoting cap-dependent translation of ISGs [35]. We therefore evaluated the impact of MEKi on the phosphorylation of eIF4E at position S209 (Fig. 6b Additional file 1: Figure S6b). There was no modulation observable due to either RSVA2 or RV2 infection compared to untreated controls (lane 5 vs lane 1) or even poly(I:C) stimulation (lane 2 vs lane 1 at 1h; lane 6 vs lane 5 at 4 h), suggesting that translation of IFN genes in AECs is not dependent on phosphorylation of eIF4E. Interestingly, MEK inhibition was not associated with decreased phosphorylation of eIF4E, indicating that MEK signaling is in fact dispensable for regulating eIF4E functionality.

In parallel to the 4E-BP1/eIF4E axis, the PI3K-AKT-mTOR pathway activates p70 S6 kinase (p70S6K) through phosphorylation of threonine 389 (T389) which is essential for its activity [36]. Interestingly, type I IFN-induced activation of PI3K-AKT pathway results in the activation of p70S6K and translation of select ISGs such as ISG15 [37]. We therefore sought to determine whether MEKi had an effect of p70S6K activation (Fig. 6c and Additional file 1: Figure S6c) and expression of *ISG15* mRNA (Fig. 6d).

Similar to the expression of 4E-BP1 following viral infections, there was no clear pattern in the activation of p70S6K at T389 after 24h infection (Fig. 6c and Additional file 1: Figure S6c). However, by looking at earlier time points following MEK inhibition and TLR3 activation, we found a significant increase in p70S6K activation in presence of MEKi (Fig. 6c, right panel, lane 4 vs lane 1; 2.4-fold at 1h, *p* < 0.05; Additional file 1: Figure S6c). MEKi-induced increase activation of p70S6K was associated with elevated expression of *ISG15* mRNA in RV2-infected (4.3-fold, *p* < 0.05) but not in RSVA2-infected cells (Fig. 6d), highlighting again the ability of RSVA2 to counteract the positive effect generated by MEKi. In the poly(I:C) model (Additional file 1: Figure S6d), there was a significant upregulation of *ISG15* mRNA expression in cells in MEKi/poly(I:C) treated cells compared to poly(I:C) controls by 24h p.t. (1.5-fold, *p* < 0.05). In accordance with the literature, our data show an association between 4E-BP1 and p70S6K axis and type I IFN response.

### Coincidently, MEKi reduces expression of IFNB1 gene repressor, PRDI-BF1, by dampening STAT3 activation but this is an RSVA2 specific effect

**Fig. 7:**
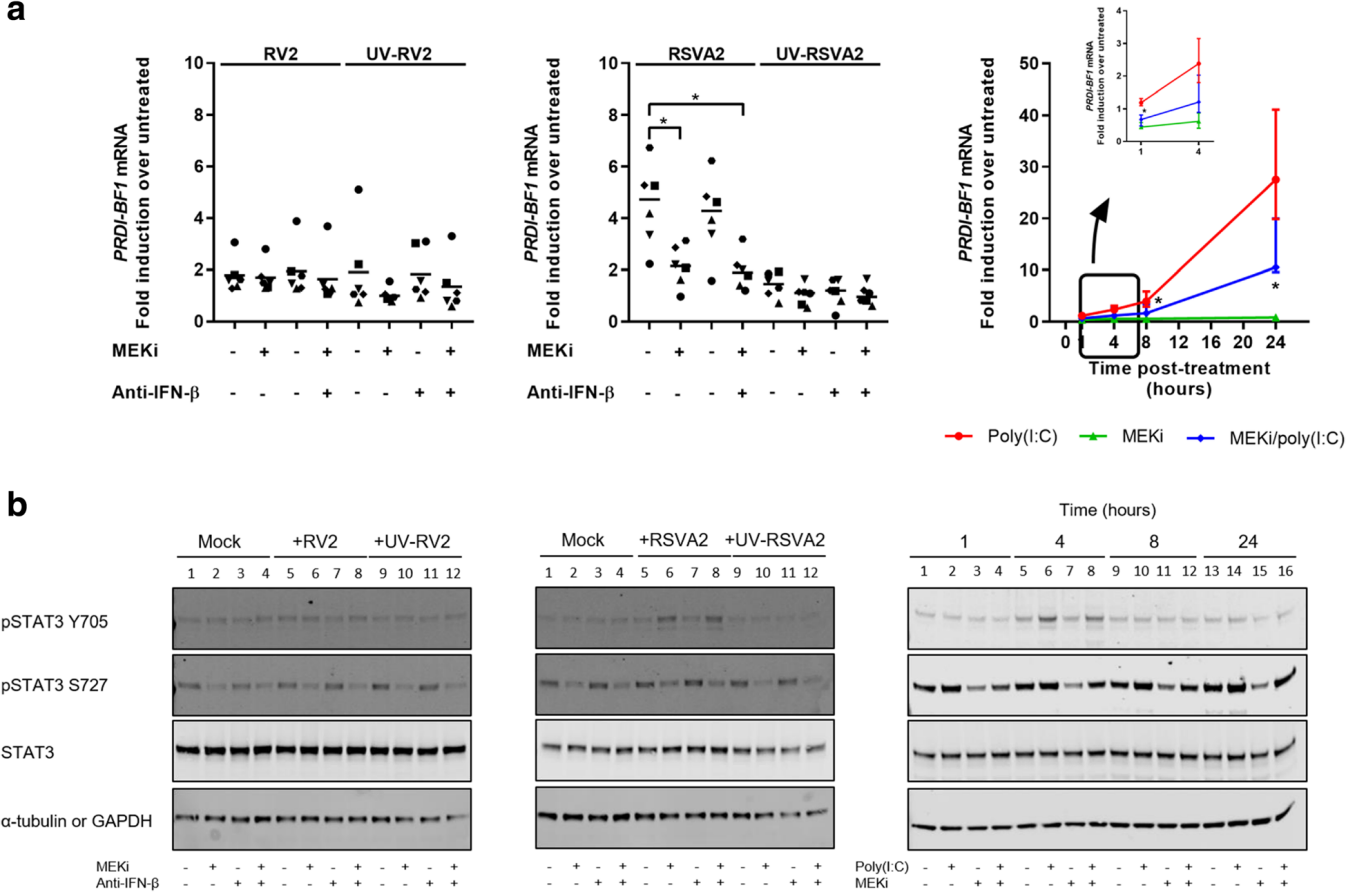
Coincidently, MEKi reduces expression of IFNB1 gene repressor, PRDI-BF1, by dampening STAT3 activation but this is an RSVA2 specific effect. **a** qRT-PCR analysis of *PRDI-BF1* mRNA in AECs pretreated with DMSO, MEKi, or anti-IFN-β antibody for 1h followed by infection with or without RV2 or RSVA2 or UV-inactivated viruses for 24h (MOI 0.1). For the poly(I:C) model (right panel), qRT-PCR analysis of *PRDI-BF1* mRNA was assessed in AECs pretreated with DMSO or MEKi for 1h and subsequently stimulated with or without poly(I:C) for 1-24h. **b** Immunoblot analysis (with anti-pSTAT3s, anti-STAT3 and anti-α-tubulin) of AECs pretreated with DMSO, MEKi, or anti-IFN-β antibody for 1h followed by infection with or without RV2 or RSVA2 or UV-inactivated viruses for 24h (MOI 0.1). For the poly(I:C) model (right panel), immunoblot analysis of AECs pretreated with DMSO or MEKi for 1h and subsequently stimulated with or without poly(I:C) for 1-24h. Each symbol () represents a donor and the horizontal bars represent the grand median in a (*n* = 6). Data are presented as median ± interquartile range in **a** (poly(I:C) model, *n* = 6). **b** is a representative immunoblot (*n* = 6). Statistical analysis was performed with the Wilcoxon signed-rank test. * *p* < 0.05 indicates statistical significance

We investigated the expression of a known postinduction repressor of *IFNB1* gene, positive regulatory domain I − binding factor1 (PRDI-BF1) that directly competes with IRF3 for DNA binding [38] (Fig. 7a). RSVA2 induced higher *PRDI-BF1* mRNA expression compared to RV2 (3-fold, *p* < 0.05), and this is commensurate to the expression of IFNs as RSVA2 induced greater response compared to RV2 (Fig. 2a). Importantly, MEKi reduced the expression of *PRDI-BF1* mRNA in RSVA2-infected cells compared to RSVA2 alone (0.5-fold, *p* < 0.05) (Fig. 7a, middle panel). However, MEKi did not affect the expression of *PRDI-BF1* mRNA in RV2-infected cells (Fig. 7a, left panel), which might be explained by a weaker induction of the *IFNB1* gene by RV2 compared to RSVA2 (Fig. 2a). In the poly(I:C) model, MEKi also caused a reduction in cells co-treated with poly(I:C) compared to poly(I:C) alone (0.6-fold at 1h, *p* < 0.05, 0.6-fold at 4h, *p* = 0.094, 0.4-fold at 8h, *p* < 0.05, 0.4-fold at 24 h, *p* < 0.05) (Fig. 7a, right panel).

To better define the effects of MEKi on PRDI-BF1 expression, we measured activation of STAT3, which was shown to be at the center of the ERK-PRDI-BF1 axis [39, 40] (Fig. 7b). Cytokines and growth factors lead to the phosphorylation of STAT3 at Y705 and the transcriptional activity of STAT3 is further enhanced by phosphorylation at S727 in an ERK dependent manner [41, 42].

Both RV2 and RSVA2 significantly induced phosphorylation of STAT3 at Y705 in presence of MEKi compared to untreated by 24h p.i. (Fig. 7b, left and middle panel, lane 6 vs lane 1; 1.5-fold, *p* < 0.05; 2-fold, *p* < 0.05; respectively; Additional file 1: Figure S7a) but not without inhibitor. However, MEKi decreased phosphorylation of STAT3 at S727 in response to RV2 or RSVA2 infection compared to untreated controls (lane 6 vs lane 1; 0.4-fold, *p* < 0.05; 0.5-fold, *p* < 0.05; respectively; Additional file 1: Figure S7b) or virus infected alone (Fig. 7b, left and middle panel, lane 6 vs lane 5; 0.5-fold, *p* < 0.05; 0.4-fold, *p* < 0.05; respectively; Additional file 1: Figure S7b), confirming the utmost importance of ERK1/2 in fully activating STAT3. The poly(I:C) model partly recapitulated the results observed with the virus experiment. By 4h p.t., poly(I:C) or MEKi/poly(I:C) comparably induced phosphorylation of STAT3 at Y705 (Fig. 7b, right panel; Additional file 1: Figure S7a). However, a striking difference was noticeable regarding STAT3 phosphorylation at S727. Similarly to the virus experiment, MEKi alone clearly decreased pSTAT3 at S727 irrespective of the time point (Fig. 7b, right panel, lane 3 vs lane 1, 0.5-fold at 1h, *p* < 0.05; lane 7 vs lane 5, 0.4-fold at 4h, *p* < 0.05; lane 11 vs lane 9, 0.5-fold at 8h, *p* < 0.05; lane 15 vs lane 13, 0.4-fold at 24h, *p* < 0.05; Additional file 1: Figure S7b). However, in presence of poly(I:C), there was significant reduction only at early time points (lane 4 vs lane 1, 0.6-fold at 1h, *p* < 0.05; lane 8 vs lane 5, 0.7-fold at 4h, *p* < 0.05; Additional file 1: Figure S7b). Conversely, poly(I:C) alone induced phosphorylation of STAT3 at S727 after 4 h (1.4-fold at 4h *p* < 0.05, 1.5-fold at 8h, *p* < 0.05, 1.9-fold at 24h, *p* < 0.05, Additional file 1: Figure S7b), suggesting that viral factors might modulate activation of STAT3 at S727. In addition, these results indicate that STAT3 phosphorylation at S727 might occur in an ERK1/2-independent manner as suggested previously [43]. More importantly, these data demonstrate that IFN signaling may be partly regulated, particularly in the late phase, by the MEK-STAT3-PRDI-BF1 axis.

## Discussion

The airway epithelium is the major target for respiratory viruses and provides a first line of defence against these pathogens. RV2 enters cells after binding to its cognate receptor (LDL) and is subsequently delivered into early endosomes where its genome is detected by TLR3. RSV, an enveloped virus, directly releases its genetic material into the cytoplasm where it activates the viral sensor RIG-I. Both pathways though converge at IRF3, which is essential in mounting an antiviral response against RSV [44] and RV [45], mainly through the production of IFN-β. Here we describe a molecular mechanism by which IRF3-driven IFN response is regulated by MEK and AKT signaling pathways in primary human AECs. The increased IFN response following MEK inhibition is driven by two separate events that are virus-dependent and occur at early and late phase: firstly, a translational derepression of *IFNB1* gene enhances IFN-β production at early stage and induce subsequent positive effects on ISGs expression. Secondly, a reduction in the expression of the transcriptional repressor PRDI-BF1 of *IFNB1* gene at later stage, when the magnitude of IFN response is higher, that further fuels the IFN-β response.

Previous studies reported a possible link between MEK inhibition and an increased type I IFN response, although these studies were performed in different cancer cell lines that exhibited constitutive type I IFN activity [46, 47]. On the other hand, viral infections activately promote the synthesis of IFNs and the expression of related genes, and therefore translation control represents a key process that ultimately determines the efficiency of the epithelial antiviral response. Several studies indeed implicated the role of signaling pathways in modulating the activation of factors linked to the translational machinery in the context of innate immunity. Type I IFN-induced PI3K-AKT pathway directly impacts the initiation of translation by hyperphosphorylating (inactivating) 4E-BP1 repressor and phosphorylating p70S6K [32, 33, 48].

Our data (Fig. 3d) and other reports [6] clearly indicate that the PI3K pathway is required for eliciting an IFN response. However, this pathway also serves viral replication purposes. Although both RSVA2 and RV2 induce a rapid activation of PI3K-AKT at early stage of infection, the purpose of this pathway differs according to the infecting virus. PI3K-AKT in RSV-infected cells promotes cell survival, thus allowing more time for the virus to replicate [49], whereas in RV-infected cells, it contributes to virus internalization [50]. In our study, we couldn’t detect activation of AKT by 24h p.t. following either RSVA2 or RV2 infection as this time point is likely to be associated with late stage phase. Despite elevated activation of AKT due to MEK inhibition, it did not support increased viral replication. The absence of antiviral activity against RSVA2 could be explained by the effects of nonstructural proteins (NS1 and 2) produced by RSVA2. IFN synthesis is compromised by multiple mechanisms including the degradation of TNF receptor-associated factor 3, an essential mediator of TLR3 signaling cascade [51], the sequestration of IRF3 [52], and the inhibition of RIG-I-MAVS interaction [53]. Remarkably, the impact of MEKi overcame the ability of RSVA2 to suppress IFN synthesis, suggesting that MEKi relieves translational silencing before the production of NS proteins takes place. However, the boosted IFN production was not associated with increased expression of ISGs, indicating possible interference of viral factors with IFN signaling. Indeed, NS proteins target STAT2 and promote its degradation in an ubiquitin dependent [54] and independent manner [51].

Another point of difference between RV2 and RSVA2 is related to the intensity of the IFN response as RSVA2 was a much more robust and potent inducer of IFN response. This higher response was correlated with increased expression of the transcriptional repressor PRDI-BF1, which functions as a post-induction feedback regulator of *IFNB1* gene expression. The decreased expression of PRDI-BF1 following MEKi treatment could be explained by the absence of STAT3 activation, highlighting the importance of MEK pathway in controlling IFN response in the late phase.

Interestingly, RSVA2 antagonized the beneficial effect due MEKi on IFN-λ1 response, indicating that RSVA2 may control transcriptional regulation of *IFNL1* gene. The activation of *IFNB1* gene requires the coordinated formation of a set of factors including IRF3, ATF2/cJun, and NF-κB that form in combination the β-enhanceosome whereas that of *IFNL1* relies more on NF-κB, IRF3 and IRF1 [55, 56]. All these factors are constitutively expressed and are activated upon viral infection except for IRF1, which is induced upon IFN signaling [57]. Considering the rapidity by which translational repressor 4E-BP1 is inactivated (within an hour) and the ability of RSVA2 to negate the effects of MEKi on ISGs (i.e. IRF1), these events might partly explain the absence of positive modulation of *IFNL1* gene following RSVA2 infection.

In addition to the improved antiviral defence of AECs, MEKi showed anti-inflammatory properties through the reduction of IL-8/CXCL8 production while levels of IL-1β and IL-6 were unaffected. Our findings are consistent with previously published reports that showed the importance of ERK in inducing IL-8/CXCL8 production by AECs [58, 59]. IL-8/CXCL8 gene activation is under the control of both transcription factors NF-κB and the activator protein (AP-1), with the latter directly regulated by ERK at both transcriptional and post-translational levels [60]. An augmented innate immunity without the deleterious effects linked to inflammation may be clinically beneficial for individuals prone to respiratory viral infections.

In this study, we used undifferentiated cells, which may not be physiologically representative of the airway epithelium in the airways. The culture of AECs at air-liquid interface will allow for differentiation into ciliated apical, basal and mucus-producing goblet cells, and thus is a more clinically relevant model. Both RV and RSV can infect basal cells from the pseudostratified epithelium, which resemble the phenotype of undifferentiated cells [61–63]. Interestingly, basal cells are more susceptible to viral infections compared to suprabasal secretory and ciliated cells, which suggest that differentiated cells are more resistant to viral infections. Our data show that RV2 load was effectively reduced in undifferentiated cells treated with MEKi, which indicate that MEKi might further improve host defence capability of well responsive differentiated cells.

A limitation worth considering in this study is the use of laboratory strains (i.e. RV2 and RSVA2), which might not be reflective of clinical isolates. Several studies showed that pathogenicity of clinical isolates differ from laboratory strains [64, 65] indicating that epithelial antiviral response may be strain-specific. In addition, there is also a differential virulence amongst RV serotypes, particularly serotype B which is notably less virulent compared to RV-A or RV-C [66, 67]. Comparing the antiviral properties of MEKi against RV clinical isolates from each serogroup may be important to consider in future studies.

There is currently no approved treatment for RV infection or available vaccine and modulating host defence of cells fighting viral infections may represent a pertinent strategy for not only decreasing burden of RV infections but also reducing exacerbations episodes in asthmatic individuals. Recently, a clinical trial aimed to evaluate whether virus-induced asthma symptoms may be prevented or improved after the inhalation of IFN-β [68]. According to the study, RV infections accounted for more than 60% of virus-induced asthma exacerbations and there was no significant improvement in the asthma control questionnaire. A contributing factor to the trial for not meeting its primary end point might be the timing of administration (24h after reporting the cold), which indicates that IFN cannot reverse damages caused by viral infection. However, it has been reported that a prophylactic approach is more effective in preventing infection in RV-inoculated volunteers [69–71], which might give a window of opportunity for using MEKi as a prophylactic agent in susceptible individuals. Furthermore, these patients may also benefit from the anti-inflammatory effects of MEK inhibition.

The antiviral effect of MEKi may not be limited to RV as MEK has been identified as a key pathway that directly contributes to the life cycle of influenza virus by facilitating nuclear export of viral ribonucleoprotein [72] and several studies indeed showed antiviral effect of different MEK inhibitors against influenza [73–75]. Moreover, a recent report suggests that MEK pathway contributes to RSV spread by facilitating the translocation of the viral F protein to the plasma membrane, which is essential for the genesis of filament and subsequent formation of syncytium [76]. In our study, we did not observe any effect of MEKi on RSVA2 load which could be explained by technical differences (a lower MOI - 0.1 vs 1; and an earlier time point - 24 vs 48h). Also, much higher concentrations of a different MEK inhibitor have been used in the previous study [76]. Number of infected cells increases over time and the likelihood of cell-to-cell contact, critical for syncytium formation, is inevitably higher. Our study clearly shows that MEKi does not reduce RSVA2 load, indicating that direct RSVA2 release is not a MEK dependent mechanism as suggested by Chang et al in a previous study [77].

## Conclusion

**Fig. 8:**
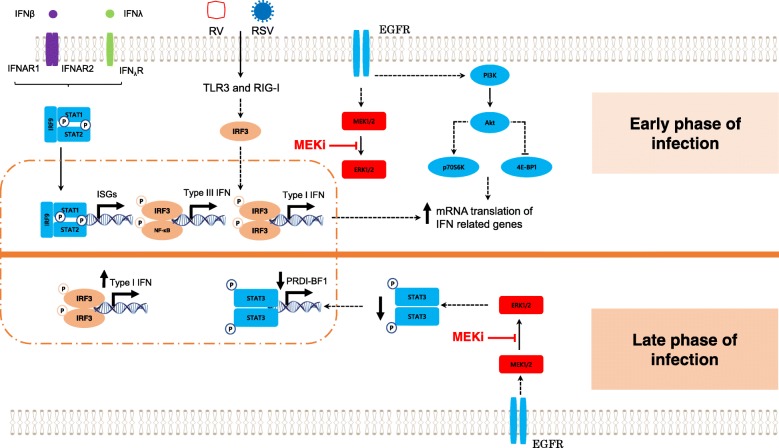
Proposed model of MEK as a key regulatory pathway of the IFN response against RV and RSV in airway epithelial cells. PI3K activation leads to activation of the AKT pathway following MEK inhibition. Upon RV or RSV infection, TLR3/RIG-I sensors activate the IRF3 pathway, which enables the transcription of type I and III IFN genes. At early stage of the infection, AKT induces deactivation of the translational repressor 4E-BP1 by phosphorylation and activation of p70S6K by phosphorylation, which eventually promote initiation of mRNA translation of IFN related genes. At late stage of a potent viral infection (RSV), MEK inhibition decreases STAT3 activation, which results in the reduction of the expression of the transcription repressor of *IFNB1* gene, PRDI-BF1, thereby creating a supplementary positive feedback loop

Based on our findings and data from previously published studies, we proposed a model by which MEK pathway controls innate antiviral response in human airway epithelial cells (Fig. 8). Inhibition of MEK activates the PI3K-AKT pathway, which in turn relieves translational silencing of *IFNB1* and *IFNL1* genes and relevant ISGs. In addition, MEK inhibition may further amplify the IFN response by reducing expression of transcriptional repressor, PRDI-BF1, if cells are exposed to a more prolonged and/or intense viral challenge. In conclusion, targeting the MEK pathway may represent a viable strategy for the development of a broad antiviral agent against several important respiratory viruses.

## Additional file

Additional file 1: ** Figure S1.** MEK inhibitor works to reduce pERK1/2 and enhances IFN-β release. **Figure S2.** Challenge with poly(I:C) is similar but not identical to challenge with RV2. **Figure S3.** PI3Ki does not cause cytotoxicity. **Figure S4.** Enhanced IFN-β response due to MEKi is not associated with increased IRF7 protein expression. **Figure S5.** Challenge with poly(I:C) is similar but not identical to challenge with RV2 or RSVA2. **Figure S6.** Induction of ISG15 mRNA by poly(I:C) is similar to that of RV2. **Figure S7.** MEKi reduces phosphorylation of STAT3 at S727. **Table S1.** NHBE donors. **Table S2.** Structure of small molecules and PROTACs. **Table S3.** List of antibodies used for immunoblotting. **Table S4.** Target sequence of short interfering RNA (siRNA). **Table S5.** List of Taqman primer/probe. (DOCX 1110 kb)

## Data Availability

All data were generated or analyzed during this study are included in this published article.

## Abbreviations

4E-BP1: Eukaryotic translation initiation factor 4E-binding protein 1
AECs: Airway epithelial cells
AKT: Protein kinase B
CPE: Cytopathic effect
EC50: Half maximal effective concentration
EGFR: Epidermal growth factor receptor
eIF4E: Eukaryotic translation initiation factor 4E
ERK: Extracellular signal-regulated kinase
HERC5: HECT and RLD domain containing E3 ubiquitin protein ligase 5
IFIT1: Interferon induced protein with tetratricopeptide repeats 1
IFN: Interferon
IFNAR: IFN-α receptor
IKKε: Inhibitor-κB kinase ε
IRF3: Interferon regulatory factor 3
ISGF3: IFN-stimulated gene factor 3
ISGs: Interferonstimulated genes
JAK1: Janus kinase 1
LDH: Lactate dehydrogenase
MDA5: Melanoma differentiation-associated gene 5
MEK: Dual specificity mitogen-activated protein kinase kinase
mTOR: mammalian target of rapamycin
MX1: Myxovirus resistance 1
p70S6K: p70 S6 kinase
PI3K: Phosphoinositide-3-kinase
PRDI-BF1: Positive regulatory domain I-binding Factor1
PROTAC: Proteolysis targeting chimeras
PRR: Pattern recognition receptor
RAF: Rapidly accelerated fibrosarcoma
RANTES/CCL5: Regulated upon activation, normal T cell expressed, and secreted / chemokine ligand 5
RIG-I: Retinoic acid inducible gene I
RSV: Respiratory syncytial virus
RV: Rhinovirus
SOCS: Suppressor of cytokine signaling
STAT: Signal transducer and activator of transcription
TBK1: Tank binding kinase 1
TLR3: Toll-like receptor 3
TYK2: Tyrosine kinase 2
USP18: Ubiquitin specific peptidase 18
