# Supplementary material for: MEK inhibition drives anti-viral defence in RV but not RSV challenged human airway epithelial cells through AKT/p70S6K/4E-BP1 signalling
Source: Cell Commun Signal. 2019 Jul 18;17:78. doi: 10.1186/s12964-019-0378-7 (PMC6639958; doi:10.1186/s12964-019-0378-7)
Supplement: Supplementary file 1 — Figure S1. MEK inhibitor works to reduce pERK1/2 and enhances IFN-β release. Figure S2. Challenge with poly(I:C) is similar but not identical to challenge with RV2. Figure S3. PI3Ki does not cause cytotoxicity. Figure S4. Enhanced IFN-β response due to MEKi is not associated with increased IRF7 protein expression. Figure S5. Challenge with poly(I:C) is similar but not identical to challenge with RV2 or RSVA2. Figure S6. Induction of ISG15 mRNA by poly(I:C) is similar to that of RV2. Figure S7. MEKi reduces phosphorylation of STAT3 at S727. Table S1. NHBE donors. Table S2. Structure of small molecules and PROTACs. Table S3. List of antibodies used for immunoblotting. Table S4. Target sequence of short interfering RNA (siRNA). Table S5. List of Taqman primer/probe. (DOCX 1110 kb) [file 12964_2019_378_MOESM1_ESM.docx]

**Additional file 1**

**Figure S1. MEK inhibitor works to reduce pERK1/2 and enhances IFN-β release**

**
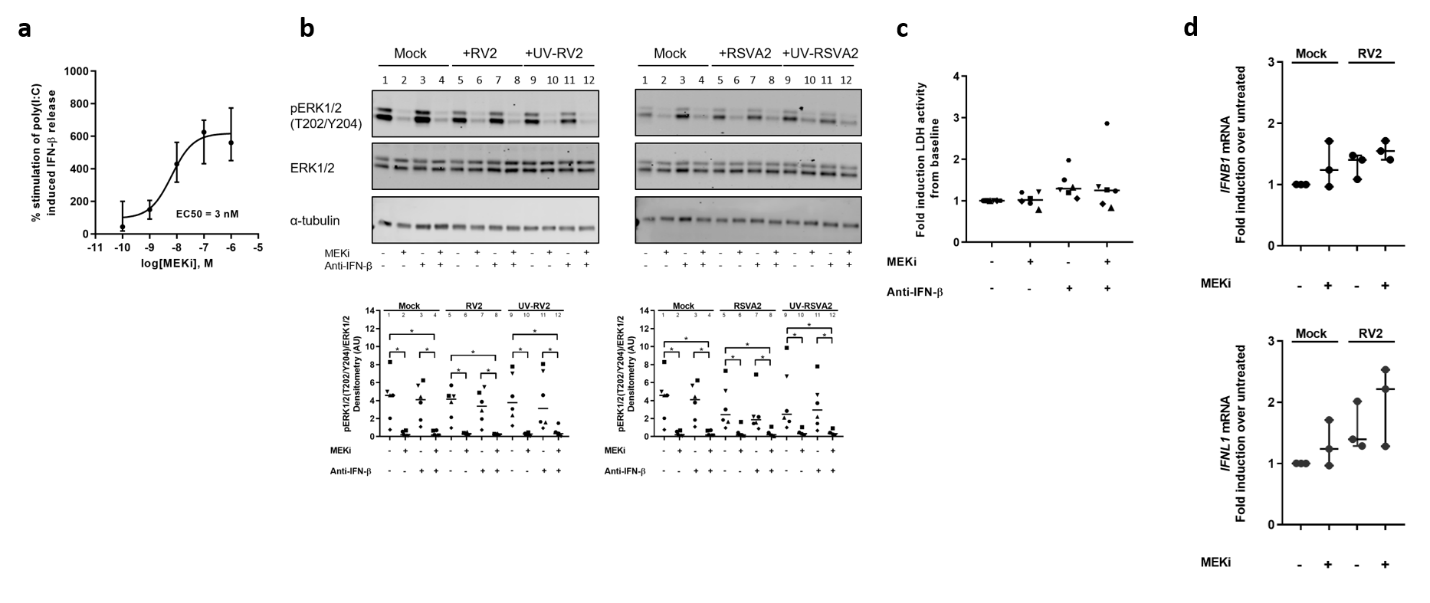
**

(a) AlphaLISA analysis of IFN-β in the supernatants of AECs pretreated with MEKi at the indicated concentration and EC50 determined accordingly. (b) Immunoblot analysis (with anti-pERK1/2, anti-ERK1/2 and anti-α-tubulin) of AECs pretreated with DMSO, MEKi, or anti-IFN-β antibody for 1h followed by infection with or without RV2 or RSVA2 or UV-inactivated viruses for 24h (MOI 0.1). Densitometry quantification of the ratio of phospho/total ERK1/2 from immunoblots. Each band was normalized to loading control α-tubulin. (c) Cytotoxicity analysis of LDH release in the supernatants of AECs pretreated with DMSO, MEKi, or anti-IFN-β antibody(d) qRT-PCR analysis of *IFNB1* and *IFNL1* mRNA in H1-Hela cells infected with RV2 for 24h (MOI 0.1). Data are presented as median ± interquartile range in a (n=6). b consists of representative immunoblots (n=6). Each symbol (
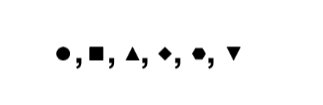
) represents a donor and the horizontal bars represent the grand median in b and c. Three independent experiments in d. Statistical analysis was performed with the Wilcoxon signed-rank test. **p*< 0.05 indicates statistical significance.

**Figure S2.** **Challenge with poly(I:C) is similar but not identical to challenge with RV2**

**
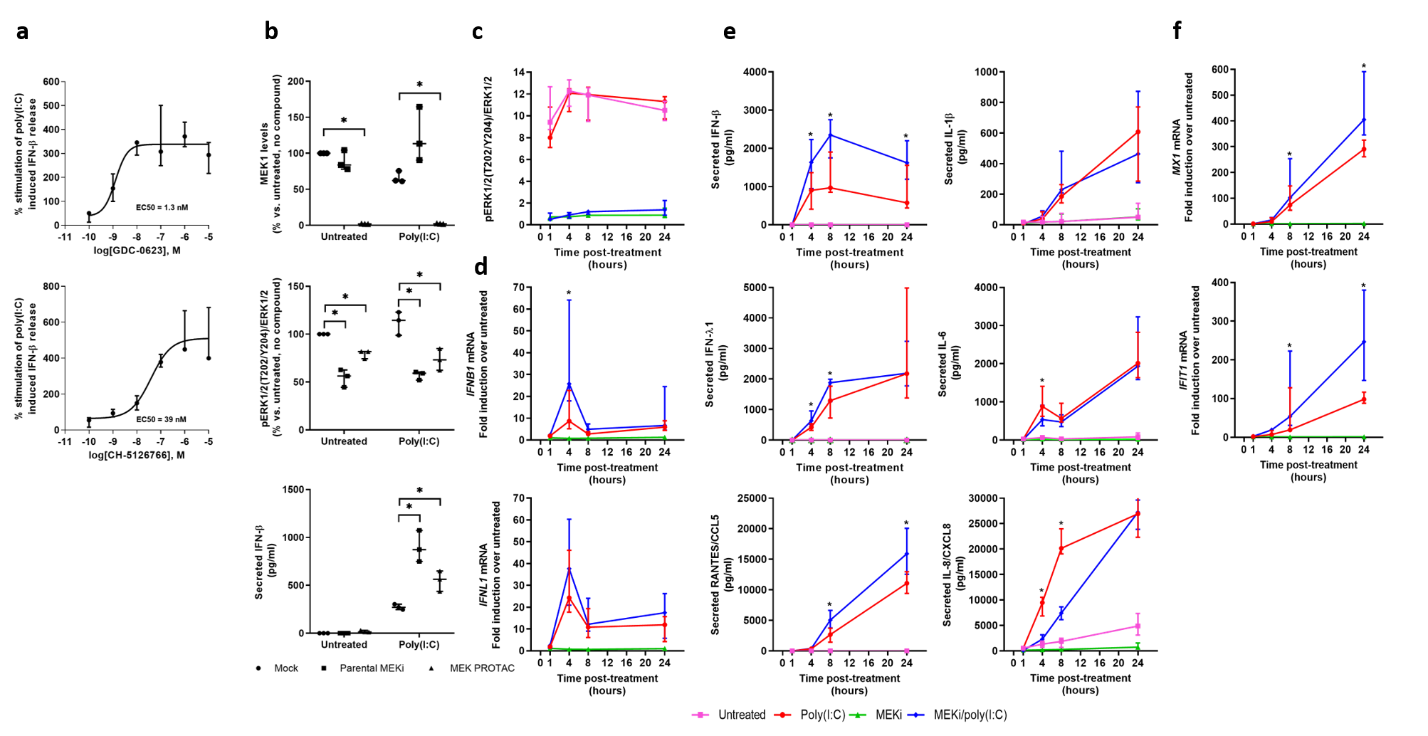
**

(a) AlphaLISA analysis of IFN-β in the supernatants of AECs pretreated with different MEK inhibitors at the indicated concentration and EC50 determined accordingly. (b) BEAS-2B cells were treated for 16 hours with 10 µM of either the parental small molecule MEK inhibitor (parental MEKi) or the MEK PROTAC, with DMSO as vehicle control. Then poly(I:C) was added for additional 4 hours and MEK1 and phospho-ERK1/2 levels determined by SureFire^®^ assays. Supernatants were collected for IFN-β measurement by AlphaLISA. (c) Simultaneous quantification of pERK1/2 / total ERK1/2 was determined in AECs pretreated with DMSO or MEKi for 1h and subsequently stimulated with or without poly(I:C) for 1-24h. (d) qRT-PCR analysis of *IFNB1* or *IFNL1* mRNA was assessed in AECs pretreated with DMSO or MEKi for 1h and subsequently stimulated with or without poly(I:C) for 1-24h. (e) AlphaLISA analysis of IFN-β, RANTES/CCL5, IL-1β, IL-6, and IL-8/CXCL8 was assessed in the supernatants of AECs pretreated with DMSO or MEKi for 1h and subsequently stimulated with or without poly(I:C) for 1-24h. IFN-λ1 production was measured by ELISA. (f) qRT-PCR analysis of *Mx1* and *IFIT1* mRNA was assessed in AECs pretreated with DMSO or MEKi for 1h and subsequently stimulated with or without poly(I:C) for 1-24h.

Data presented as median ± interquartile range in a, c, d, e and f (n=6). Three independent experiments in b. Statistical analysis was performed with the Wilcoxon signed-rank test. **p* < 0.05 indicates statistical significance.

**Figure S3. PI3Ki does not cause cytotoxicity**


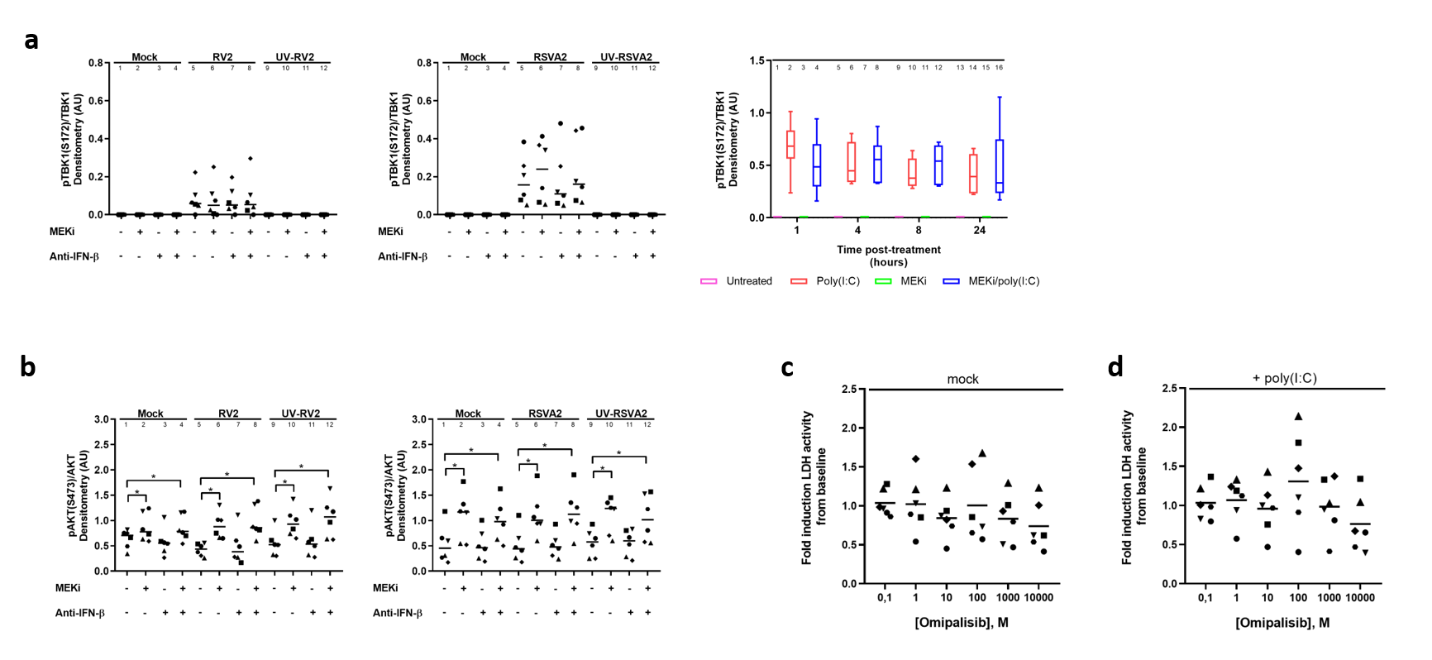


(a) Densitometry quantification of the ratio of phospho/total TBK1 from immunoblots from Fig. 3a. Each band was normalized to loading control α-tubulin. (b) Densitometry quantification of the ratio of phospho/total AKT from immunoblots from Fig. 3b. (c, d) Cytotoxicity analysis of LDH release in the supernatants of AECs pretreated with PI3Ki, for 1h and subsequently stimulated with or without poly(I:C) for 4h.

Each symbol (
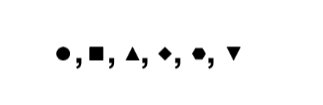
) represents a donor and the horizontal bars represent the grand median (n=6). On each box plot (poly(I:C) model), the central bar represents the median and the whiskers represent minimum and maximum (n=6). Statistical analysis was performed with the Wilcoxon signed-rank test. **p*< 0.05 indicates statistical significance.

**Figure S4. Enhanced IFN-β response due to MEKi is not associated with increased IRF7 protein expression**


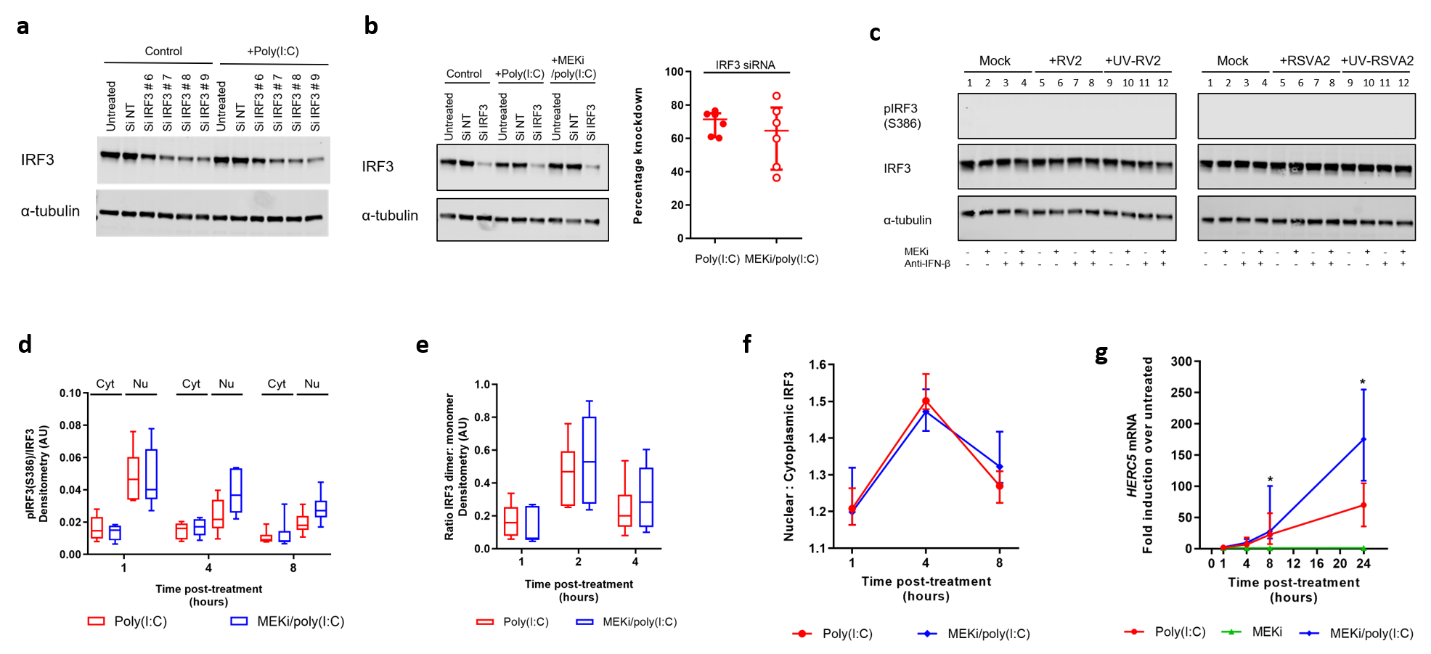


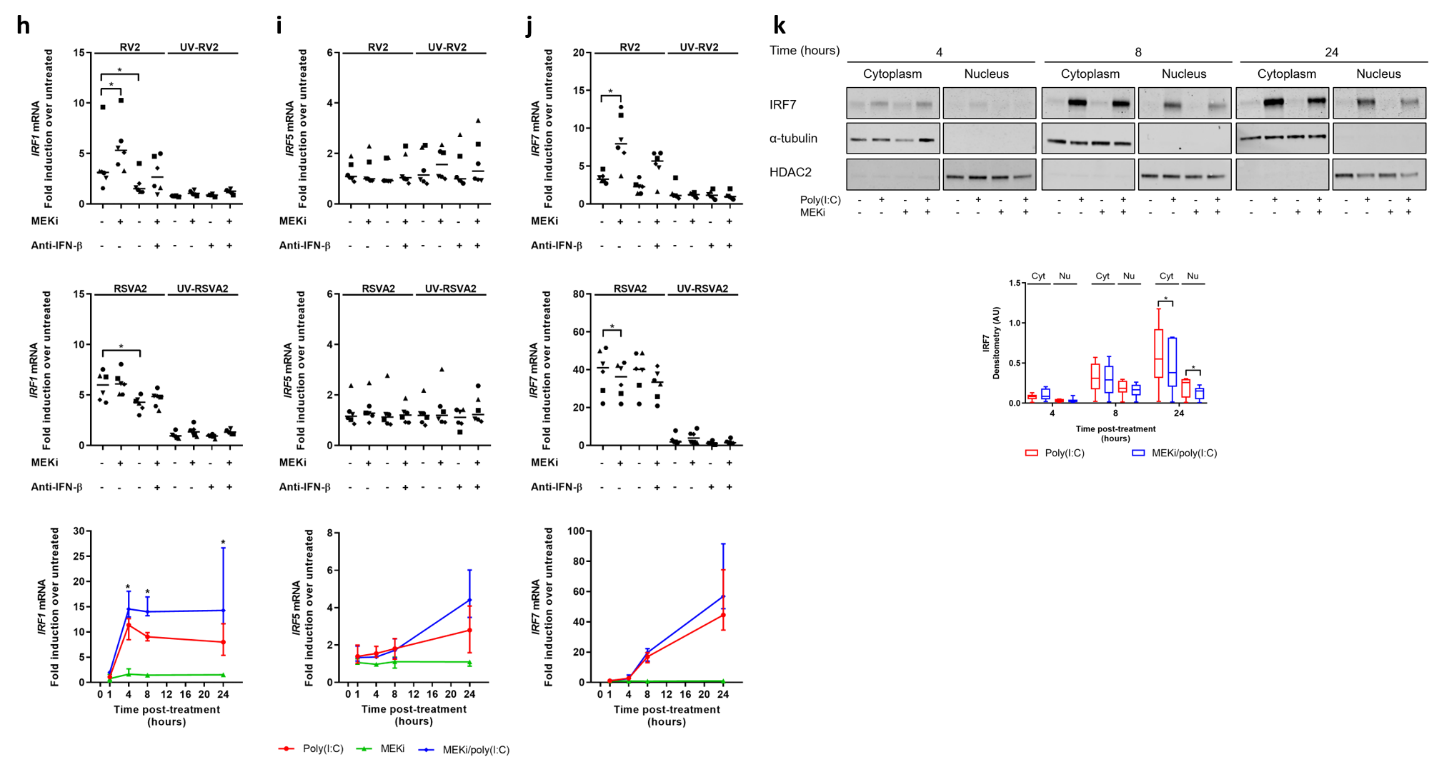


(a) Immunoblot analysis (with anti-IRF3 and anti-α-tubulin) of AECs transfected with IRF3 or NT siRNA followed by stimulation with DMSO or poly(I:C) for 4h. (b) Immunoblot analysis (with anti-IRF3 and anti-α-tubulin) of AECs transfected with IRF3 (#7) or NT siRNA followed by treatment with DMSO or MEKi and subsequent stimulation with or without poly(I:C) for 4h. Percentage knockdown was determined accordingly. (c) Immunoblot analysis (with anti-pIRF3, anti-IRF3 and anti-α-tubulin) of AECs pretreated with DMSO, MEKi, or anti-IFN-β antibody for 1h followed by infection with or without RV2 or RSVA2 or UV-inactivated viruses for 24h (MOI 0.1). (d) Densitometry quantification of the ratio of phospho/total IRF3 from Fig. 4b. Each band was normalized to loading control α-tubulin or HDAC2. (e) Densitometry quantification of the ratio of IRF3 dimer:monomer from Fig. 4c . Each band was normalized to total protein levels. (f) Nuclear to cytoplasmic ratio of IRF3 in AECs pretreated with MEKi and subsequently stimulated with poly(I:C) for 1-8h. (g) qRT-PCR analysis of *HERC5* mRNA was assessed in AECs pretreated with DMSO or MEKi for 1h and subsequently stimulated with or without poly(I:C) for 1-24h. (h) qRT-PCR analysis of *IRF1* mRNA in AECs pretreated with DMSO, MEKi, or anti-IFN-β antibody for 1h followed by infection with or without RV2 or RSVA2 or UV-inactivated viruses for 24h (MOI 0.1). For poly(I:C) model (lower panel), qRT-PCR analysis of *IRF1* mRNA was assessed in AECs pretreated with DMSO or MEKi for 1h and subsequently stimulated with or without poly(I:C) for 1-24h. (i) qRT-PCR analysis of *IRF5* mRNA in AECs pretreated with DMSO, MEKi, or anti-IFN-β antibody for 1h followed by infection with or without RV2 or RSVA2 or UV-inactivated viruses for 24h (MOI 0.1). For poly(I:C) model (lower panel), qRT-PCR analysis of *IRF5* mRNA was assessed in AECs pretreated with DMSO or MEKi for 1h and subsequently stimulated with or without poly(I:C) for 1-24h. (j) qRT-PCR analysis of *IRF7* mRNA in AECs pretreated with DMSO, MEKi, or anti-IFN-β antibody for 1h followed by infection with or without RV2 or RSVA2 or UV-inactivated viruses for 24h (MOI 0.1). For poly(I:C) model (lower panel), qRT-PCR analysis of *IRF7* mRNA was assessed in AECs pretreated with DMSO or MEKi for 1h and subsequently stimulated with or without poly(I:C) for 1-24h. (k) Immunoblot analysis (with anti-IRF7, α-tubulin, and HDAC2) of fractionated AECs pretreated with DMSO or MEKi for 1h followed by stimulation with or without poly(I:C) for 4-24h. Densitometry quantification of IRF7. Each band was normalized to loading control α-tubulin or HDAC2.

a, b, c, and k are representative immunoblots (n=6). Data are presented as median ± interquartile range in b, f, g, h, i, and j (n=6). d, e, and k are box plots and the central bar represents the median and the whiskers represent minimum and maximum (n=6).

Each symbol (
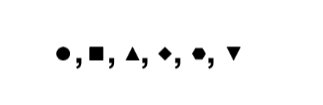
) represents a donor and the horizontal bars are the grand median in h, i, and j (RV2 and RSVA2) (n=6). Statistical analysis was performed with the Wilcoxon signed-rank test. **p*< 0.05 indicates statistical significance.

**Figure S5.** **Challenge with poly(I:C) is similar but not identical to challenge with RV2 or RSVA2**


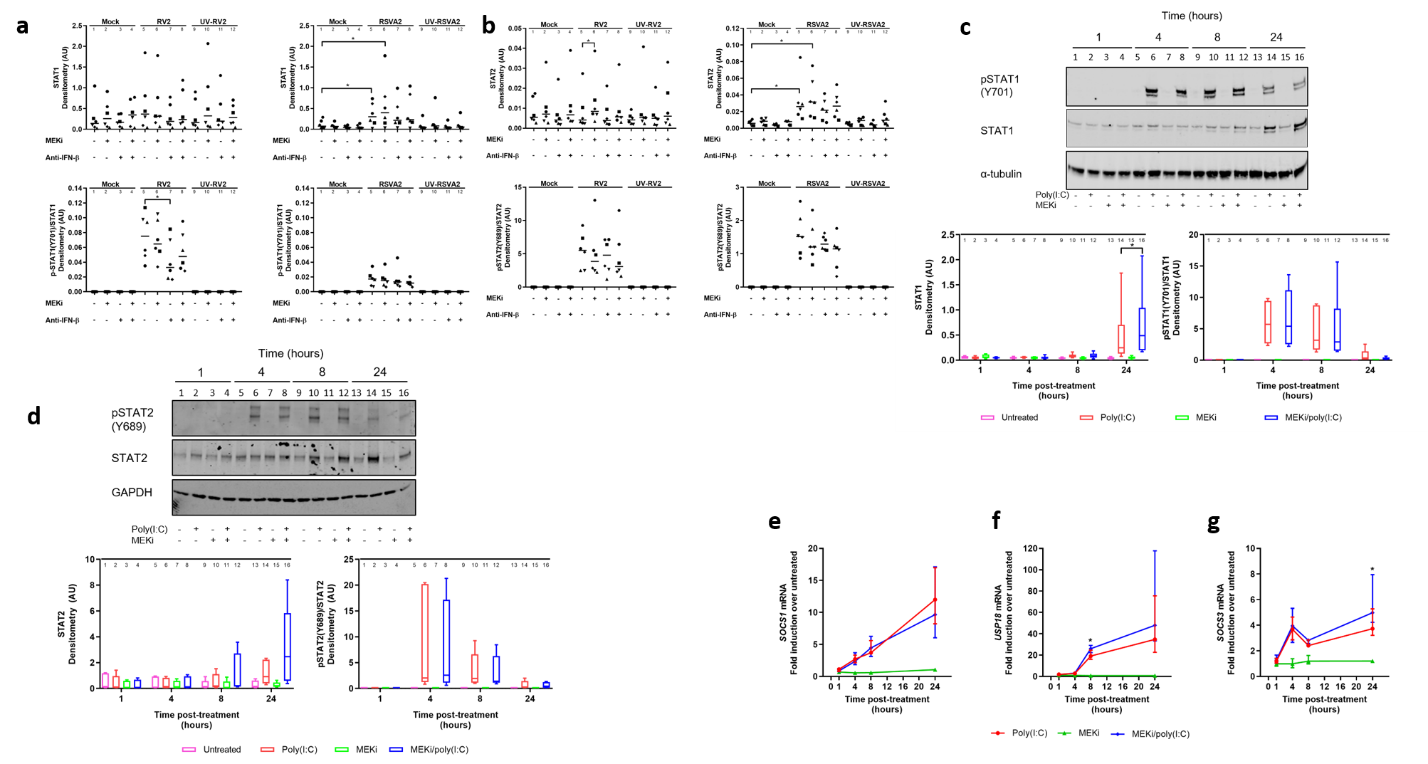


(a) Densitometry quantification of STAT1 and the ratio of phospho/total STAT1 from immunoblots from Fig. 5a. (b) Densitometry quantification of STAT2 and the ratio of phospho/total STAT2 from immunoblots from Fig. 5b. (c) Immunoblot analysis (with anti-pSTAT1, anti-STAT1 and α-tubulin) of AECs pretreated with DMSO or MEKi for 1h and subsequently stimulated with or without poly(I:C) for 1-24h. Densitometry quantification of STAT1 and the ratio of phospho/total STAT1 from immunoblots. (d) Immunoblot analysis (with anti-pSTAT2, anti-STAT2 and α-tubulin) of AECs pretreated with DMSO or MEKi for 1h and subsequently stimulated with or without poly(I:C) for 1-24h. Densitometry quantification of STAT2 and the ratio of phospho/total STAT2 from immunoblots. (e) qRT-PCR analysis of *SOCS1* mRNA was assessed in AECs pretreated with DMSO or MEKi for 1h and subsequently stimulated with or without poly(I:C) for 1-24h. (f) qRT-PCR analysis of *USP18* mRNA was assessed in AECs pretreated with DMSO or MEKi for 1h and subsequently stimulated with or without poly(I:C) for 1-24h. (g) qRT-PCR analysis of *SOCS3* mRNA was assessed in AECs pretreated with DMSO or MEKi for 1h and subsequently stimulated with or without poly(I:C) for 1-24h.

Each symbol (
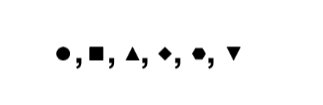
) represents a donor and the horizontal bars represent the grand median in a and b (n=6). c and d are representative immunoblots (n=6). On each box plot in c and d, the central bar represents the median and the whiskers represent minimum and maximum (n=6). Data are presented as median ± interquartile range in e, f, and g (n=6). Statistical analysis was performed with the Wilcoxon signed-rank test. **p* < 0.05 indicates statistical significance.

**Figure S6. Induction of *ISG15* mRNA by poly(I:C) is similar to that of RV2**


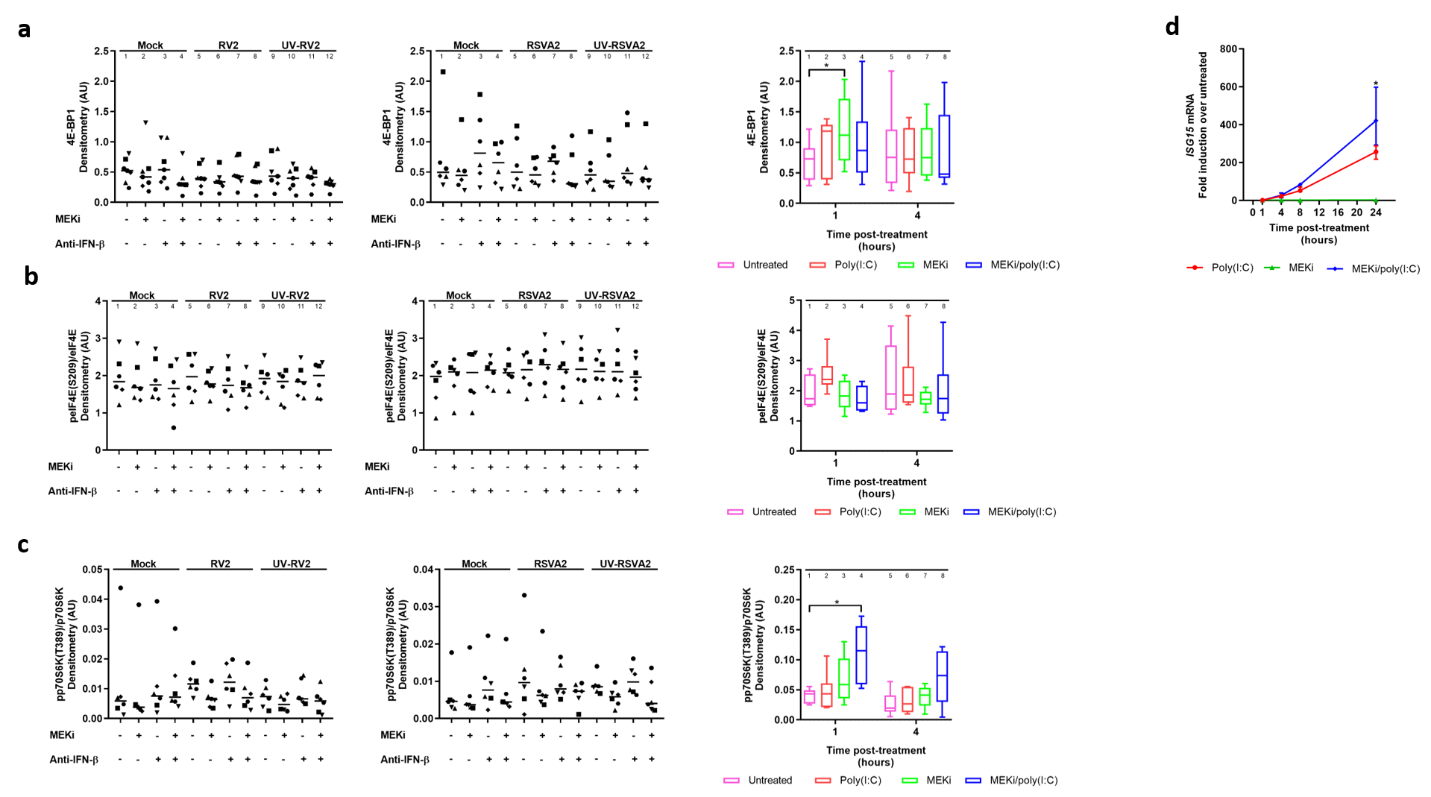


(a) Densitometry quantification of 4E-BP1 from immunoblots from Fig. 6a. (b) Densitometry quantification of the ratio of phospho/total eIF4E from immunoblots from Fig. 6b. (c) Densitometry quantification of the ratio of phospho/total p70S6K from immunoblots from Fig. 6c. (d) qRT-PCR analysis of *ISG15* mRNA was assessed in AECs pretreated with DMSO or MEKi for 1h and subsequently stimulated with or without poly(I:C) for 1-24h.

Each symbol (
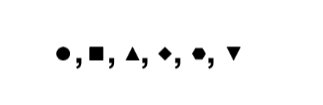
) represents a donor and the horizontal bars represent the grand median in a, b, and c (n=6). On each box plot in a, b, and c (poly(I:C) model), the central bar represents the median and the whiskers represent minimum and maximum (n=6). Data are presented as median ± interquartile range in d. Statistical analysis was performed with the Wilcoxon signed-rank test. **p*< 0.05 indicates statistical significance.

**Figure S7. MEKi reduces phosphorylation of STAT3 at S727**


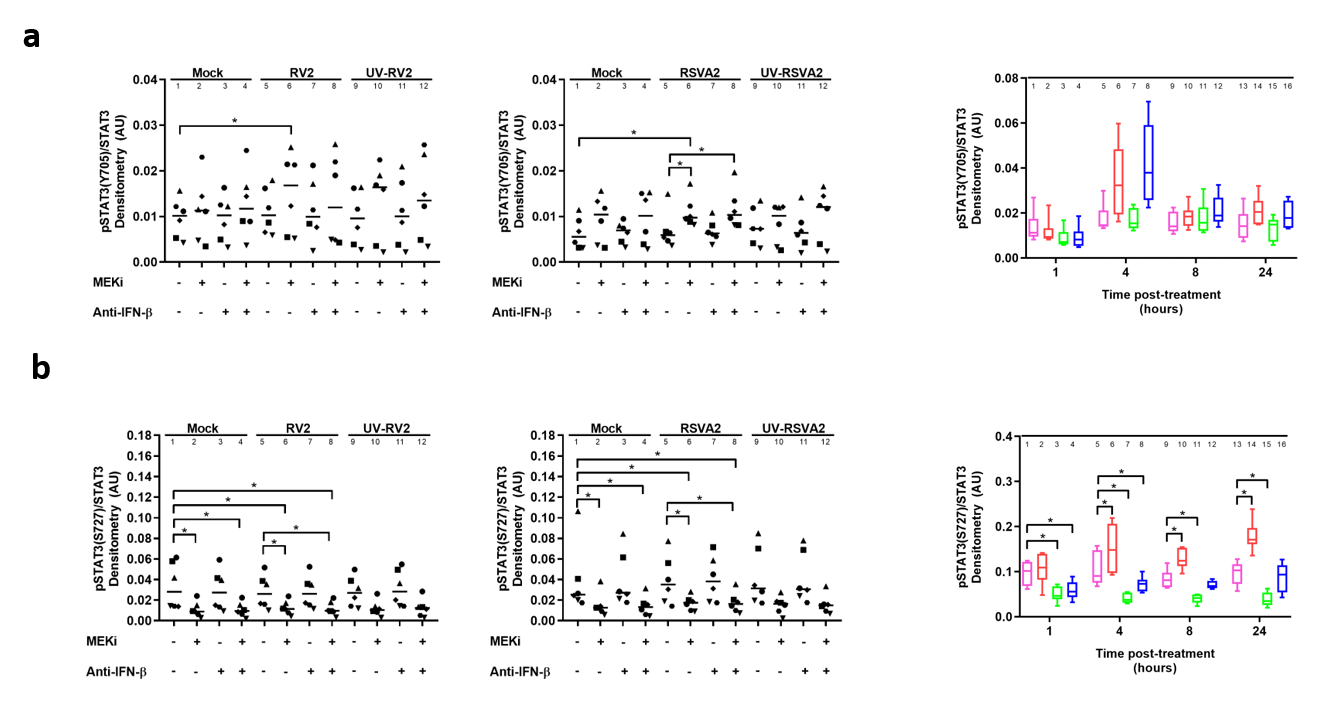


(a and b) Densitometry quantification of the ratio of phospho/total STAT3 from immunoblots from Fig. 7a and b. Each symbol (
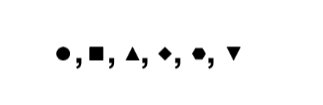
) represents a donor and the horizontal bars are the grand median in a and b (n=6). On each box plot in a and b (poly(I:C) model), the central bar represents the median and the whiskers represent minimum and maximum (n=6). Statistical analysis was performed with the Wilcoxon signed-rank test. **p*< 0.05 indicates statistical significance.

**Table S1. NHBE donors**

| **Donor #** | **Age** | **Sex** |
| --- | --- | --- |
| 0000485960 | 38 | M |
| 0000448571 | 28 | M |
| 0000627466 | 24 | F |
| 0000444771 | 69 | M |
| 0000489938 | 2 | F |
| 0000613375 | 65 | F |

**Table S2. Structure of small molecules and PROTACs**

| **Name** | **IUPAC Name** | **Mode Of Action** | **Structure** | **IC^50 c^** | **EC^50^ value for poly(I:C) induced IFN-β release** |
| --- | --- | --- | --- | --- | --- |
| **PD-0325901** | N-[(2R)-2,3-dihydroxypropoxy]-3,4-difluoro-2-(2-fluoro-4-iodoanilino)benzamide | Allows MEK1/2 to be phophorylated by RAF, promotes complex formation between RAF and MEK (Type I). Suppresses phosphorylation of downstream effector ERK | 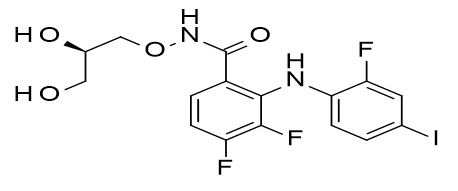 | 1 nM for MEK1/2 (1) | 3 nM |
| **GDC-0623** | 5-(2-fluoro-4-iodoanilino)-N-(2-hydroxyethoxy)imidazo[1,5-a]pyridine-6-carboxamide | Inhibits MEK1/2 phosphorylation by RAF (Type III) | 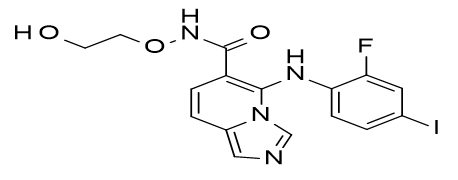 | N.D. (2) | 1.3 nM |
| **CH-5126766** | 3-[[3-fluoro-2-(methylsulfamoylamino)pyridin-4-yl]methyl]-4-methyl-7-pyrimidin-2-yloxychromen-2-one | Inhibits MEK1/2 phosphorylation by RAF and stabilises an inactive conformation of RAF and MEK (Type III) | 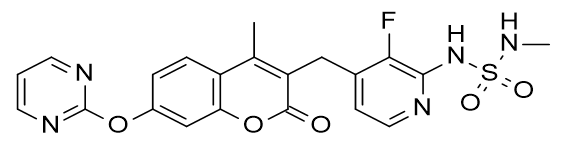 | 160 nM for MEK1 (3) | 39 nM |
| **Compound 29** | (2S,4R)-1-((S)-2-(tert-butyl)-18-(4-(N-(2-((2-fluoro-4-iodophenyl)amino)-1,5-dimethyl-6-oxo-1,6-dihydropyridin-3-yl)sulfamoyl)phenyl)-4,16-dioxo-6,9,12-trioxa-3,15-diazaoctadecanoyl)-4-hydroxy-N-((S)-1-(4-(4-methylthiazol-5-yl)phenyl)ethyl)pyrrolidine-2-carboxamide | MEK PROTAC induces degradation of MEK1 & 2 through VHL mediated ubiquitination | 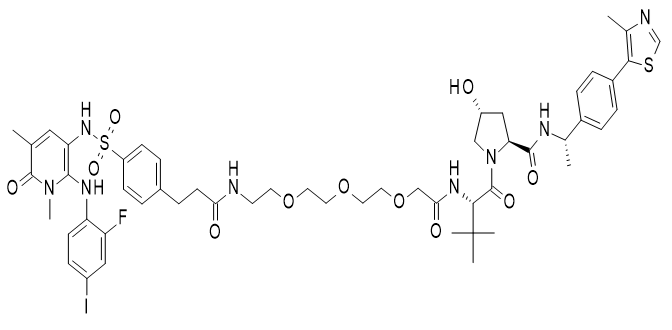 | DC50 MEK1 ~500 nM (Vollmer et al., 2019 under preparation) | n.d. |
| **GSK2126458** | 2,4-difluoro-N-{2-methoxy-5-[4-(pyridazin-4-yl)quinolin-6-yl]pyridin-3-yl}benzene-1-sulfonamide | Binds to and inhibits PI3K | 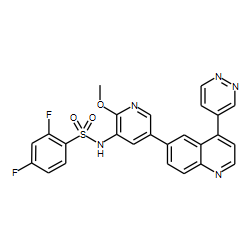 | 0.04 nM for PI3Kα (4) | NA |
| **AZ14118425-001; SN1062730994 (parental compound)** | N-[2-(2-fluoro-4-iodo-anilino)-1,5-dimethyl-6-oxo-3-pyridyl]benzenesulfonamide | ATP-noncompetitive inhibitor of MEK kinase activity | 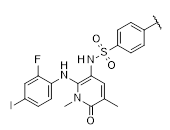 | 10 nM (Vollmer et al., 2019 under preparation) | n.d. |

References

1. Sebolt-Leopold JS, Herrera R. Targeting the mitogen-activated protein kinase cascade to treat cancer. Nat Rev Cancer. 2004;4(12):937-47. Epub 2004/12/02. doi: 10.1038/nrc1503. PubMed PMID: 15573115.
2. Hatzivassiliou G, Haling JR, Chen H, Song K, Price S, Heald R, et al. Mechanism of MEK inhibition determines efficacy in mutant KRAS- versus BRAF-driven cancers. Nature. 2013;501(7466):232-6. Epub 2013/08/13. doi: 10.1038/nature12441. PubMed PMID: 23934108.
3. Ishii N, Harada N, Joseph EW, Ohara K, Miura T, Sakamoto H, et al. Enhanced inhibition of ERK signaling by a novel allosteric MEK inhibitor, CH5126766, that suppresses feedback reactivation of RAF activity. Cancer Res. 2013;73(13):4050-60. Epub 2013/05/15. doi: 10.1158/0008-5472.CAN-12-3937. PubMed PMID: 23667175; PubMed Central PMCID: PMCPMC4115369.
4. Knight SD, Adams ND, Burgess JL, Chaudhari AM, Darcy MG, Donatelli CA, et al. Discovery of GSK2126458, a Highly Potent Inhibitor of PI3K and the Mammalian Target of Rapamycin. ACS Med Chem Lett. 2010;1(1):39-43. Epub 2010/04/08. doi: 10.1021/ml900028r. PubMed PMID: 24900173; PubMed Central PMCID: PMCPMC4007793.

**Table S3. List of antibodies used for immunoblotting**

| **Antibody** | **Source** | **Catalogue number** |
| --- | --- | --- |
| Rabbit polyclonal anti-pERK1/2 (T202/Y204) | Cell Signaling Technology | 9101 |
| Rabbit polyclonal anti-ERK1/2 | Cell Signaling Technology | 9102 |
| Mouse monoclonal anti-α-tubulin | Sigma-Aldrich | T9026 |
| Mouse monoclonal anti-GAPDH | Sigma-Aldrich | G8795 |
| Rabbit polyclonal anti-pIRF3 (S386) | Merck Millipore | ABE501 |
| Rabbit polyclonal anti-IRF3 | Proteintech | 11312-1-AP |
| Rabbit polyclonal anti-IRF7 | Proteintech | 22392-1-AP |
| Mouse monoclonal anti-HDAC2 | Cell Signaling Technology | 5113 |
| Rabbit polyclonal anti-pTBK1 (S172) | Cell Signaling Technology | 5483 |
| Rabbit polyclonal anti-TBK1 | Cell Signaling Technology | 3504 |
| Rabbit polyclonal anti-pAKT (S473) | Cell Signaling Technology | 4060 |
| Rabbit polyclonal anti-AKT | Cell Signaling Technology | 2938 |
| Rabbit polyclonal anti-4E-BP1 | Cell Signaling Technology | 9644 |
| Rabbit polyclonal anti-peIF4E (S209) | ThermoFisher Scientific | 44-528G |
| Rabbit polyclonal anti-eIF4E | Cell Signaling Technology | 9742 |
| Rabbit polyclonal anti-pp70S6K (T389) | Cell Signaling Technology | 9234 |
| Rabbit polyclonal anti-p70S6K | Cell Signaling Technology | 9202 |
| Rabbit polyclonal anti-pSTAT3 (Y705) | Cell Signaling Technology | 9131 |
| Rabbit polyclonal anti-pSTAT3 (S727) | Cell Signaling Technology | 9134 |
| Rabbit polyclonal anti-STAT3 | Cell Signaling Technology | 4904 |
| Rabbit polyclonal anti-pSTAT1 (Y701) | Cell Signaling Technology | 7649 |
| Rabbit polyclonal anti-STAT1 | Cell Signaling Technology | 9172 |
| Rabbit polyclonal anti-pSTAT2 (Y689) | Sigma | 07-224 |
| Rabbit polyclonal anti-STAT2 | Cell Signaling Technology | 72604 |
| Rabbit polyclonal anti-GAPDH | Cell Signaling Technology | 2118 |
| Goat anti-Rabbit IgG (H+L) Cross-Adsorbed Secondary Antibody, Alexa Fluor 594 | ThermoFisher Scientific | A-11012 |
| IRDye^®^ 680RD Goat anti-Mouse IgG | LI-COR Biosciences | 926-68070 |
| IRDye^®^ 800CW Goat anti-Rabbit IgG | LI-COR Biosciences | 925-32211 |

**Table S4. Target sequence of short interfering RNA (siRNA)**

| **Gene** | **Target sequence** | **Catalogue number (Dharmacon)** |
| --- | --- | --- |
| IRF3 siRNA (#6) target sequence: | CGAGGCCACUGGUGCAUAU | J-006875-06 |
| IRF3 siRNA (#7) target sequence: | CCAGACACCUCUCCGGACA | J-006875-07 |
| IRF3 siRNA (#8) target sequence: | GGAGUGAUGAGCUACGUGA | J-006875-08 |
| IRF3 siRNA (#9) target sequence: | AGACAUUCUGGAUGAGUUA | J-006875-09 |
| ON-TARGETplus Nontargeting  Pool, 5 nmol | NA | D-001810-10-05 |

**Table S5. List of Taqman primer/probe**

| **Gene** | **Catalogue number** | **Assay ID** |
| --- | --- | --- |
| Actin-b (TaqMan™Gene Expression Assay (FAM) | Cat # 4351370 | hs01060665_g1 |
| GAPDH (TaqMan™Gene Expression Assay (FAM) | Cat # 4351370 | Hs02786624_g1 |
| IRF7 (TaqMan™Gene Expression Assay (FAM) | Cat # 4351370 | Hs01014809_g1 |
| IRF5 (TaqMan™Gene Expression Assay (FAM) | Cat # 4351370 | Hs00158114_m1 |
| IRF1 (TaqMan™Gene Expression Assay (FAM) | Cat # 4351370 | Hs00971965_m1 |
| PRDI-BF1 (TaqMan™Gene Expression Assay (FAM) | Cat # 4351370 | Hs00153357_m1 |
| ISG15 (TaqMan™Gene Expression Assay (FAM) | Cat # 4351370 | Hs01921425_s1 |
| HERC5 (TaqMan™Gene Expression Assay (FAM) | Cat # 4351370 | Hs00180943_m1 |
| SOCS1 (TaqMan™Gene Expression Assay (FAM) | Cat # 4351370 | [Hs00705164_s1](https://www.thermofisher.com/taqman-gene-expression/product/Hs00705164_s1?CID=&ICID=&subtype=) |
| SOCS3 (TaqMan™Gene Expression Assay (FAM) | Cat # 4351370 | [Hs02330328_s1](https://www.thermofisher.com/taqman-gene-expression/product/Hs02330328_s1?CID=&ICID=&subtype=) |
| USP18 (TaqMan™Gene Expression Assay (FAM) | Cat # 4351370 | [Hs00276441_m1](https://www.thermofisher.com/taqman-gene-expression/product/Hs00276441_m1?CID=&ICID=&subtype=) |
| IFNb1 (TaqMan™Gene Expression Assay (FAM) | Cat # 4351370 | [Hs01077958_s1](https://www.thermofisher.com/taqman-gene-expression/product/Hs01077958_s1?CID=&ICID=&subtype=) |
| Mx1 (TaqMan™Gene Expression Assay (FAM) | Cat # 4351370 | [Hs00895608_m1](https://www.thermofisher.com/taqman-gene-expression/product/Hs00895608_m1?CID=&ICID=&subtype=) |
| IFNL1 (TaqMan™Gene Expression Assay (FAM) | Cat # 4351370 | [Hs00601677_g1](https://www.thermofisher.com/taqman-gene-expression/product/Hs00601677_g1?CID=&ICID=&subtype=) |
